# Phosphorylation of the HP1β hinge region sequesters KAP1 in heterochromatin and promotes the exit from naïve pluripotency

**DOI:** 10.1093/nar/gkab548

**Published:** 2021-07-02

**Authors:** Weihua Qin, Enes Ugur, Christopher B Mulholland, Sebastian Bultmann, Irina Solovei, Miha Modic, Martha Smets, Michael Wierer, Ignasi Forné, Axel Imhof, M Cristina Cardoso, Heinrich Leonhardt

**Affiliations:** Faculty of Biology, Ludwig-Maximilians-Universität München, Butenandtstraße 1, D-81377 Munich, Germany; Faculty of Biology, Ludwig-Maximilians-Universität München, Butenandtstraße 1, D-81377 Munich, Germany; Department of Proteomics and Signal Transduction, Max Planck Institute for Biochemistry, Am Klopferspitz 18, 82152 Martinsried, Germany; Faculty of Biology, Ludwig-Maximilians-Universität München, Butenandtstraße 1, D-81377 Munich, Germany; Faculty of Biology, Ludwig-Maximilians-Universität München, Butenandtstraße 1, D-81377 Munich, Germany; Faculty of Biology, Ludwig-Maximilians-Universität München, Butenandtstraße 1, D-81377 Munich, Germany; The Francis Crick Institute and UCL Queen Square Institute of Neurology, London NW1 1AT, United Kingdom; Faculty of Biology, Ludwig-Maximilians-Universität München, Butenandtstraße 1, D-81377 Munich, Germany; Department of Proteomics and Signal Transduction, Max Planck Institute for Biochemistry, Am Klopferspitz 18, 82152 Martinsried, Germany; Biomedical Center Munich, Faculty of Medicine, Ludwig-Maximilians-Universität München, Großhaderner Str. 9, 82152 Planegg-Martinsried, Germany; Biomedical Center Munich, Faculty of Medicine, Ludwig-Maximilians-Universität München, Großhaderner Str. 9, 82152 Planegg-Martinsried, Germany; Cell Biology and Epigenetics, Department of Biology, Technical University of Darmstadt, 64287 Darmstadt, Germany; Faculty of Biology, Ludwig-Maximilians-Universität München, Butenandtstraße 1, D-81377 Munich, Germany

## Abstract

Heterochromatin binding protein HP1β plays an important role in chromatin organization and cell differentiation, however the underlying mechanisms remain unclear. Here, we generated *HP1β^−/−^* embryonic stem cells and observed reduced heterochromatin clustering and impaired differentiation. We found that during stem cell differentiation, HP1β is phosphorylated at serine 89 by CK2, which creates a binding site for the pluripotency regulator KAP1. This phosphorylation dependent sequestration of KAP1 in heterochromatin compartments causes a downregulation of pluripotency factors and triggers pluripotency exit. Accordingly, *HP1β*^−/−^ and phospho-mutant cells exhibited impaired differentiation, while ubiquitination-deficient *KAP1^−/−^* cells had the opposite phenotype with enhanced differentiation. These results suggest that KAP1 regulates pluripotency via its ubiquitination activity. We propose that the formation of subnuclear membraneless heterochromatin compartments may serve as a dynamic reservoir to trap or release cellular factors. The sequestration of essential regulators defines a novel and active role of heterochromatin in gene regulation and represents a dynamic mode of remote control to regulate cellular processes like cell fate decisions.

## INTRODUCTION

Heterochromatin binding protein HP1 is a non-histone chromosomal protein and has a function in the establishment and maintenance of higher-order chromatin structures and gene silencing (1,2). In mammals, there are three homologues of HP1, termed HP1α, HP1β and HP1γ, encoded by *Cbx5*, *Cbx1* and *Cbx3* genes, respectively. HP1 homologues contain two conserved functional domains, an N-terminal chromodomain (CD) and a C-terminal chromoshadow domain (CSD), linked by a hinge region. The CD domain is responsible for recognition of di- and trimethylated K9 of histone H3 (H3K9me2 and H3K9me3) (3–5), while the CSD domain mediates interactions with other proteins (6,7). The intrinsically disordered regions (IDRs) and posttranslational modifications are likely responsible for the unique functions of HP1 homologues.

Recent studies testing the capacity of HP1 to induce phase separation revealed that only HP1α formed phase-separated droplets (8,9). This phase separation correlates with the formation of heterochromatin compartments (chromocenters) in the nucleus. Recently, we found that the charge of the hinge IDR (IDR-H) is a distinctive feature of HP1 homologues and plays a decisive role in liquid-liquid phase separation (LLPS) (10,11) and that HP1β also undergoes phase separation in a histone H3K9me3 dependent manner (11). HP1α/β together with other chromatin binding proteins, such as SUV39H1 and KAP1, coalesce around the solid chromatin scaffold (12–15).

In cells, HP1α and HP1γ locate at condensed heterochromatin and euchromatin, respectively, while HP1β accumulates mostly at condensed heterochromatin and to less extent at euchromatin (16). The specific functions of HP1 proteins in chromatin organization correlate with their unique cellular roles during cell differentiation. HP1β knockout mice die perinatally and show impaired development of the cerebral neocortex and neuromuscular junctions (17). In mouse embryonic stem cells (mESCs), depletion of HP1β affects differentiation (18). However, how HP1β regulates cell differentiation is unclear.

To address this question, we generated *HP1β*^−/−^ mESCs. These cells showed impaired naïve pluripotency exit and are defective in neural progenitor cell differentiation. We found that HP1β is phosphorylated at the serine 89 residue of the hinge region by casein kinase 2 (CK2). This phosphorylation creates a specific binding site for KAP1, which leads to sequestration of this pluripotency factor and downregulation of pluripotency genes. While phase separation and the formation of membraneless compartments has been implicated in the local enrichment of factors involved in the same cellular process, the sequestration of KAP1 represents a novel mechanism of transcriptional regulation and cell fate decision by remote controlled functional depletion.

## MATERIALS AND METHODS

### Cell culture, transfection and inhibitor treatment

Human embryonic kidney (HEK) 293T cells and baby hamster kidney (BHK) cells were cultured in DMEM supplemented with 10% fetal calf serum (FCS) and 50 μg/ml gentamicin (PAA).

Naive E14 mESCs (19) were cultured as described previously (20). In brief, cells were kept under naive conditions in N2B27 medium consisting of 50% DMEM/F12 (Life Technologies) supplemented with N2 (Life Technologies) and 50% neurobasal medium (Life Technologies) supplemented with serum-free B27 (Life Technologies), 2 mM l-glutamine (Life Technologies), 100 U/ml penicillin, 100 μg/ml streptomycin (PAA Laboratories GmbH) and 0.1 mM β-mercaptoethanol (Life Technologies). Naive mESCs were maintained on flasks treated with Geltrex (Life Technologies) diluted 1:100 in DMEM/F12 (Life Technologies) in N2B27 media containing 2i (1 μM PD032591 and 3 μM CHIR99021 (Axon Medchem, Netherlands)), 1000 U/ml recombinant leukemia inhibitory factor (LIF, Millipore) and 0.3% BSA (Gibco).

For the metastable state culture of mESCs, cells were cultured in gelatinized flasks in DMEM supplemented with 16% FCS, 0.1 mM β-mercaptoethanol (Invitrogen), 2 mM l-glutamine, 1× MEM non-essential amino acids, 100 U/ml penicillin, 100 μg/ml streptomycin (PAA) and 1000 U/ml recombinant leukemia inhibitory factor LIF (Millipore). For CRISPR-assisted cell line generation, the culture medium was supplemented with 2i.

To differentiate ESCs from naive to epiblast state, cells were plated on flasks treated with Geltrex (Life Technologies) in defined medium containing 20 ng/ml Activin A (R&D Systems), 10 ng/ml FGF2 (R&D Systems) and 0.1× Knockout Serum Replacement (Life Technologies). Media was changed after 24 h and epiblast cells were imaged at 48 h.

Mouse ESCs were transfected with Lipofectamine 3000 Reagent (Invitrogen) according to the manufacturer's instructions. HEK 293T and BHK cells were transfected using polyethylenimine (PEI) as transfection reagent (Sigma-Aldrich) according to the manufacturer's instructions. Cell fixation and microscopy were carried out as described (21).

To inhibit CK2 activity, 50 μM of the specific inhibitor 4,5,6,7-tetrabromobenzotriazole (TBB) was added directly after transfection. To check HP1β-pS89 levels in wt and CK2a1^as^ cells, 10 μM of the adenine analog 1-NA-PP1 was supplemented to the medium overnight.

### CRISPR/Cas-mediated gene editing and generation of stable cell lines

For generation of *HP1β*^−/−^ mESCs, the MINtag strategy was used as described previously (11,22). After generation of the MIN-tagged line, the attB-RFP-Stop-PolyA was inserted into the N-terminus of the endogenous *HP1β*^attP/attP^ locus by Bxb1 mediated recombination.

For generation of the CK2a1^as^ cell line, genome editing was performed with slight modifications compared to a previous publication (23). Briefly, the two gRNAs for editing CK2a1 were designed using the CRISPR design tool from the Zhang Lab (MIT, www.genome-engineering.org), and got incorporated into the pSpCas9 (BB)-2A-GFP (px458) vector by BpiI restriction sites (23). To mutate CK2a1 at aa position 113 from phenylalanine to alanine, a 200 nt ssDNA donor oligo was synthesized by Integrated DNA Technologies (IDT). A HpyCH4V cutting site was incorporated into the donor oligo for screening. Mouse ESCs were transfected with the Cas9-gRNA vector and a donor oligo. 48 h after transfection, GFP positive cells were sorted using FACS and plated at clonal density. After one-week, individual clones were picked and expanded for genomic DNA isolation. The mutant clones were validated by PCR using respective primers, HpyCH4V digestion and DNA sequencing (Supplementary Figure S3E).

For generation of HP1β S89A and HP1β S89E cell lines, the specific gRNA was cloned into a vector expressing GFP and SpCas9 (px458: F. Zhang Lab). To mutate HP1β from serine (S) to alanine (A) or glutamic acid (E) in aa position 89, 200 nt ssDNA donor oligos were synthesized by Integrated DNA Technologies (IDT). For screening, the HypCH4V or HypCH4IV cutting site was incorporated into the donor oligo of S89A and S89E, respectively. The mutant lines were validated by PCR using the respective primers followed by HypCH4V or HypCH4IV (New England Biolabs) digestion and DNA sequencing. The expression of HP1β S89A and HP1β S89E was analyzed by western blot and immunostaining.

For generation of the *KAP1^−/−^* cell line, KAP1-specific gRNA was cloned into a puromycin-selectable vector expressing SpCas9 (px459: F. Zhang Lab). Mouse ESCs were transfected with the Cas9-gRNA vector and two days after transfection E14 mESCs were plated at clonal density in ESC media supplemented with 1 μg/ml puromycin (Gibco). Selection media was replaced by normal ESC media after 48 h and colonies were allowed to grow for a week. Single ESC colonies were transferred into 2 × 96-well plates. Selection of *KAP1^−/−^* clones was accomplished by amplifying the CRISPR/Cas targeted region via PCR followed by PstI digestion (FastDigest; Thermo Scientific). Positive clones were assessed by sanger sequencing and western blots by using antibodies against both N- and C-terminus of KAP1 (Figure 6B and Supplementary Figure S8).

To generate KAP1-GFP mESCs, gRNA specific to C-terminus of KAP1 locus was cloned into a puromycin-selectable vector expressing both SpCas9 (px459: F. Zhang Lab). mESCs were transfected with the Cas9-gRNA vector and a 719 bp donor synthesized by Integrated DNA Technologies (IDT). Two days after transfection, cells were subjected to puromycin (1 μg/ml) for two days. A week later, GFP positive cells were sorted using FACS (Supplementary Figure S10B).

For generation of stable mESC and HEK293T lines, 48 h after expression of GFP-tagged constructs (GFP-HP1β wt, GFP-HP1β S89/91A, GFP-HP1β S89/91D, GFP-KAP1 wt, GFP-KAP1 RH, GFP-KAP1 PVL, GFP-KAP1 RH/PVL), cells were plated at clonal density and subjected to blasticidin selection (10 μg/ml) for a week. Then, GFP positive cells were separated using a fluorescence activated cell sorting (FACS) Aria II instrument (Becton Dickinson).

The cell lines and expression constructs are listed in Supplementary Table S1.

### Immunofluorescence staining

For immunostaining, mESCs were grown on coverslips coated with Geltrex (Life Technologies). After rinsing coverslips 2× with PBS (pH 7.4; 140 mM NaCl, 2.7 mM KCl, 6.5 mM Na2HPO4, 1.5 mM KH_2_PO_4_), cells were fixed for 10 min with 3.7% formaldehyde (Sigma), washed 3× for 10 min with PBST (PBS, 0.01% Tween20), permeabilized for 5 min in PBS supplemented with 0.5% Triton X-100 and washed 2× for 10 min with PBS. Primary and secondary antibodies (see Supplementary Table S1) were diluted in blocking solution (PBST, 3% BSA). After the incubation steps with the respective antibody in a humidified dark chamber for 1 h, coverslips were washed 3× for 10 min with PBST. For DNA counterstaining, coverslips were incubated in a solution of DAPI (400 ng/ml) in PBS-T for 5 min, before washing 3× for 10 min with PBST. Coverslips were mounted in antifade containing medium (Vectashield, Vector Laboratories) and sealed with colorless nail polish. Images were collected using a Leica TCS SP5 confocal microscope equipped with Plan Apo 63×/1.4 NA oil immersion objective and lasers with excitation lines 405, 488, 594 and 633 nm.

### Co-immunoprecipitation and western blotting

For co-immunoprecipitation, 1 × 10^7^ mESCs were lysed in lysis buffer (10 mM Tris/Cl pH7.5, 150 mM NaCl, 0.5 mM EDTA, 0.5% NP40, 1.5 mM MgCl_2_, 0.5 U/ml Benzonase (Sigma-Aldrich), 1 mM PMSF, 1× mammalian Protease Inhibitor Cocktail (Serva^®^) at 4°C for 30 min. Lysate was cleared up by centrifugation at 20 000 × g at 4°C for 15 min and protein concentration was measured with Pierce™ 660 nm Protein Assay Reagent according to the manufacturer's instructions. Equal amounts of protein extracts were incubated with 80 μl of anti- HP1β-pS89 antibody for 2 h at 4°C under constant rotation. Then, 20 μl of protein G beads (GE) were added to the protein extracts and incubated overnight under constant rotation at 4°C. Beads were washed 3× with washing buffer (10 mM Tris/Cl pH7.5, 300 mM NaCl, 0.5 mM EDTA) and boiled in Laemmli buffer at 95°C for 10 min. Bound fractions were separated and visualized by western blotting.

For immunoprecipitation, HEK 293T cells stably expression GFP-HP1β wt and its mutants were treated with hypotonic buffer (10 mM Tris–HCl pH 8, 10 mM KCl, 1.5 mM MgCl_2_, 1 mM DTT and 1× Protease Inhibitor, 2 mM PMSF) for 30 min and centrifuged at 1000 × g at 4°C to get the intact nuclei. Nuclei were lysed in a lysis buffer at 4°C for 30 min. Lysates were first cleared by centrifugation at 20 000 × g for 15 min at 4°C and then incubated with a GFP-Trap (Chromotek). Bound fractions were visualized by a coomassie stained polyacrylamide gel.

For the general detection of HP1β on western blots, a rabbit anti-HP1β antibody (Abcam and Cell Signaling Technology, see Supplementary Table S1) was used. For the specific detection of HP1β-pS89, antibodies against the peptide GKRKADpSDSEDKG were raised in mice and rats. RFP or Cherry fusion proteins were detected by the rat-anti-red antibody 5F8 (24). KAP1 was visualized by rabbit anti-KAP1 antibodies (Abcam and Proteintech, see Supplementary Table S1). Equal loading of cell lysates was assessed by a mouse anti-β-actin antibody (Sigma-Aldrich), a mouse anti-tubulin antibody (Sigma-Aldrich) and a polyclonal H3 antibody (Abcam, see Supplementary Table S1). Secondary antibodies, anti-rabbit (Biorad), anti-rat and anti-mouse (Dianova), were conjugated to horseradish peroxidase and visualized with ECL Plus reagent (GE Healthcare, Thermo Scientific). Signals were acquired on an Amersham Imager 600 (GE).

Antibodies used in this study are listed in Supplementary Table S1.

### F3H assay

The F3H assay was performed as described previously (25). In brief, BHK cells containing lac operator arrays were transiently transfected on coverslips using PEI and fixed with 3.7% formaldehyde 16 h after transfection. For DNA counterstaining, coverslips were incubated in a solution of DAPI (400 ng/ml) in PBST and mounted in Vectashield. Images were taken using a SP5 Leica confocal microscope equipped with Plan Apo 63×/1.4 NA oil immersion objective and lasers with excitation lines: 405 nm for DAPI, 488 nm for GFP fusions, 561 nm for Cherry fusions and 633 nm for HP1β-pS89.

### Flow cytometry analysis

For flow cytometry, plates were washed once with PBS, dissociated to single cells by trypsin-EDTA treatment, resuspended in PBS buffer supplemented with 2% FBS and 1 mM EDTA, and incubated with DyLight-650-conjugated anti-SSEA-1 (clone MC-480, MA1-022-D650, Life Technologies) antibody for 30–60 min on ice. Cells were spun down, resuspended in a buffer containing DAPI for live-dead cell staining and analyzed using a FACS Aria II (BD Biosciences). Cell debris was excluded by forward and side scatter gating. FlowJo was used for data analysis.

### Mass spectrometry of in-gel digests

In-gel digests were performed according to standard protocols. Briefly, after washing the excised gel slices proteins were reduced by DTT, alkylated with iodoacetamide and digested with trypsin (Sequencing Grade Modified, Promega) overnight at 37°C. For protein identification the resulting peptides were purified on-line with C18 reversed cartridge (Dionex) and separated in an Ultimate 3000 RSLCnano system (Thermo Fisher Scientific), using in a 15-cm analytical column (75 μm ID home-packed with ReproSil-Pur C18-AQ 2.4 μm from Dr Maisch) with a 50-min gradient from 5 to 60% acetonitrile in 0.1% formic acid. The effluent from the HPLC was directly electrosprayed into Orbitrap-LTQ XL (Thermo Fisher Scientific) operated in data dependent mode to automatically switch between full scan MS and MS/MS acquisition. Survey full scan MS spectra (from *m*/*z* 300 –2000) were acquired in the Orbitrap with resolution *R* = 60 000 at *m*/*z* 400 (after accumulation to a ‘target value’ of 500 000 in the linear ion trap). The six most intense peptide ions with charge states between 2 and 4 were sequentially isolated to a target value of 10 000 and fragmented in the linear ion trap by collision induced dissociation (CID). All fragment ion spectra were recorded in the LTQ part of the instrument. For all measurements with the Orbitrap detector, 3 lock-mass ions from ambient air were used for internal calibration. Typical MS conditions were spray voltage, 1.5 kV; no sheath and auxiliary gas flow; heated capillary temperature, 200°C; normalized CID energy 35%; activation *q* = 0.25; activation time = 30 ms. Proteins were identified using Mascot (Matrix Science, London, UK; version Mascot) against SwissProt_2011.02 database for human proteins (Fragment Tolerance: 0.80 Da, Fixed Modification for carbamidomethyl cysteine, Variable Modification for methionine oxidation, Max Missed Cleavage: 2).

### Protein purification and histone isolation

HP1 cDNA was cloned into a pET28 expression vector, mutants were made using overlap extension PCR and proteins were subsequently expressed in *Escherichia coli*. Purifications of HP1β proteins were described previously (11).

KAP1 cDNA was cloned into a pCAG-GFP expression vector and respective mutants were made using overlap extension PCR. HEK293T were transfected with the plasmid coding for GFP-KAP1, harvested 48 h after transfection and lysed in lysis buffer (50 mM NaH_2_PO_4_ pH 8.0, 300 mM NaCl, 10 mM imidazole, 0,5% Tween-20, 2 mM MgCl2, 0.5 U/ml Benzonase, 1 mM PMSF, 1× mammalian protease inhibitor cocktail.) at 4°C for 30 min. Cell debris were removed by centrifugation at 20 000 × g for 15 min at 4°C. Cleared cell lysate was incubated with Ni-NTA-GBP beads for 1.5 h under constant rotation at 4°C. The beads were washed 3× with washing buffer (50 mM NaH_2_PO_4_ pH 7.5, 300 mM NaCl, 20 mM imidazole, 0.05% Tween-20) before eluting the protein with elution buffer (10 mM Tris pH 7.5, 100 mM KCl, 1 mM EDTA, 1 mM DTT and 250 mM imidazole). Protein concentration was assessed by measuring its GFP emission signal on a plate reader (TECAN) with purified GFP as standard reference.

Histone isolation was conducted as previously described with minor changes of the protocol (26). In brief, 15 × p100 HEK293T cells were harvested, and cell pellets were resuspended in a hypotonic buffer. To obtain pure nuclei, cells were disrupted using a homogenizer and nuclei were subsequently incubated in a chromatin dissociation buffer (10 Tris–HCl pH 8.0, 20 mM EDTA and 400 mM NaCl) for 30 min on ice. This chromatin dissociation step was repeated 4×. Afterwards, nuclei were resuspended in 0.4 N H2SO4 and incubated on a rotator at 4°C overnight. After centrifugation, histones in the supernatant were transferred into a fresh reaction tube and precipitated using 33% Trichloroacetic acid (TCA). After washing 3x with cold acetone, histones were dissolved in H2O and centrifuged at 2000 rpm for 5 min to remove precipitations. Histone concentrations were measured using the PierceTM 660 nm protein assay kit.

### *In vitro* droplet assays

For the droplet assay, proteins were concentrated to ∼10 μg/μl using Amicon concentrators. After the concentration step, buffer was exchanged to 20 mM HEPES pH 7.2, 75 mM KCl, 1 mM DTT with Zeba™ Spin Desalting Columns. For the spin down assay, 30 μl of turbid solution was spun down at 2000 rpm for 5 min and 29 μl of supernatant was transferred into a Protein LoBind Tube (Eppendorf). The supernatant and droplets were boiled in 120 μl laemmli buffer at 95°C for 10 min. 10 μl of supernatant and droplets were loaded into a SDS-PAGE gel for following detection via coomassie stain.

For visualization of His-tagged-HP1β within the droplets, 500 ng of protein was labeled according to the Monolith NT™ Protein Labeling Kit RED-NHS from Nano Temper. After buffer exchange, 50 ng of the labeled protein was added into the droplet solution. For visualization of GFP-KAP1 within the droplets, 100 ng of protein was incubated with HP1β at 4°C for 3 min before adding histones.

### Neuronal progenitor cell (NPC) differentiation

The differentiation of pluripotent ESCs into NPCs was based on a protocol described before (27). Simply, ESCs maintained with naïve medium (2i/LIF) were switched to the metastable culturing medium (serum/LIF) one week before the NPC differentiation. At the D0, 4 × 10^6^ cells were plated onto bacteriological Petri dishes (Greiner) in 15 ml cellular aggregates (CA) medium (DMEM supplemented with 10% FCS, 2 mM L-glutamine, 1 × non-essential amino acids and 0.1 mM β-mercaptoethanol). At the D4, 5 μM of the retinoic acid (RA) was added into the CA medium. At the D8, the CAs were dissociated with freshly prepared trypsin and were plated onto PORN/laminin-coated plated with N2 medium (125 ml DMEM, 125 ml F-12, 1.25 ml insulin (25 μg/ml), 6.25 ml transferrin (50 μg/ml), 0.25 ml progesterone (20 nM), 0.25 ml putrescine (100 nM), 25 μl sodium selenite (30 nM), 0.5× l-glutamine, 1× Pen/Strep and 50 μg/ml BSA). Samples from different time points of differentiation were collected for analyses.

### Alkaline phosphatase (AP) staining

One thousand mESCs were seeded into one well of a six-well plate and cultured for 6 days prior to the AP staining. The AP staining was performed as published previously (28) using the Alkaline Phosphatase Detection Kit (Sigma-Aldrich) according to the manufacturer's instructions.

### RNA isolation and RNA sequencing and transcriptome analysis

For RNA-seq, RNA was isolated using the NucleoSpin Triprep Kit (Machery-Nagel) according to the manufacturer's instructions. Digital gene expression libraries for RNA-seq were produced using a modified version of single-cell RNA barcoding sequencing (SCRB-seq) optimized to accommodate bulk cells (29) in which a total of 70 ng of input RNA was used for the reverse-transcription of individual samples. RNA-seq libraries were sequenced on an Illumina HiSeq 1500. The libraries were sequenced paired end with 15–20 cycles to decode sample barcodes and UMI from read 1 and 45 cycles into the cDNA fragment. Similar sequencing qualities were confirmed by FastQC v0.10.1.

To generate principal component analysis (PCA) plot, SCRB-seq pools (i7) were demultiplexed from the Illumina barcode reads using deML (30). All reads were trimmed to the same length of 45 bp by cutadapt (31) (v1.8.3) and mapped using Spliced Transcripts Alignment to a Reference (STAR) (32) and mapped to the mouse genome (mm10). Gene-wise count/UMI tables were generated using the published Drop-seq pipeline (v1.0) (33). PCA was performed on the 1000 most variable genes to display the major variance between the genotype and differentiation state.

To check gene expression during NPC differentiation, RNA-seq libraries were processed and mapped to the mouse genome (mm10) using the zUMIs pipeline (34). UMI count tables were filtered for low, plasmids, counts using HTSFilter (35). Differential expression analysis was performed in R using DESeq2 (36) and genes with an adjusted *P*<0.05 were considered to be differentially expressed.

For GO analysis of biological processes the online tool (http://cbl-gorilla.cs.technion.ac.il/) was used (37,38). For the analysis of *HP1β^−/−^* and HP1β S89A, genes showing >1.5-fold changes (Supplementary Table S2) were considered. The upregulated and downregulated genes in *KAP1*^−/−^ (Supplementary Table S4) were separately analyzed. The GO analyses were done by two unranked lists of genes with p-value thresholds of 1.0E−03 and 1.0E−05.

### Chromatin immunoprecipitation and sample preparation for mass spectrometry

Chromatin immunoprecipitation coupled to mass spectrometry (ChIP-MS) of HP1β was performed in two technical replicates for WT and HP1β-KO mESCs and EpiLCs by using a direct HP1β antibody (Abcam). For each replicate, independently grown 15 × 10^6^ cells were harvested and crosslinked as described previously (39). Next, nuclei were isolated with a mild lysis buffer (20 mM Tris–HCl pH 8.0, 85 mM KCl, 0.5% NP40, 1× PIC) and briefly pelleted for 5 min at 2000 × g and 4°C. To digest DNA, nuclei were resuspended in an MNase digestion buffer (1 M sorbitol, 50 mM Tris–HCl pH 8.0, 5 mM CaCl_2_, 1× PIC). Subsequently, 2 μl MNase (NEB, 6000 gel units) was added and, after 1 min prewarming at 37°C, samples were incubated for 12.5 min at 37°C and at 1000 rpm in a thermal shaker. The reaction was quenched by the addition of EGTA to a final concentration of 50 mM. Nuclei were then spun down and resuspended in the IP-Buffer (50 mM Tris–HCl pH 8.0, 100 mM NaCl, 5 mM EDTA, 0.3% SDS, 1.7% Triton X-100, 1× PIC). Samples were addressed to brief sonication (3 × 30 s) at low setting in a Bioruptor Plus (Diagenode). Lysates were then centrifuged for 20 min at maximum speed and 4°C. To check the DNA digestion efficiency 20 μl of each sample was diluted to 5% in TBS and 10 μl proteinase K (Invitrogen) was added. These quality check samples were incubated O/N at 65°C under constant shaking to reverse FA-crosslinks. The next day, samples were incubated with 5 μl RNaseA (10 mg/ml) and incubated for 30 min at 37°C. The DNA was purified (Quagen Quaquick PCR purification kit) and DNA sizes were checked on an 1% agarose gel.

Meanwhile samples for ChIP-MS were kept on ice. If the shearing efficiency was in the range of 150–500 bp the protein concentration of the ChIP-MS samples was estimated by a BCA assay (Thermo). Each replicate was diluted to 1 mg/ml in 1 ml total volume and 1 μg of antibody was added. The samples were incubated O/N at 4°C under constant rotation.

The next day, for each sample 20 μl (slurry volume) of magnetic protein A/G beads (Sigma) were washed 3x in the IP buffer and subsequently aliquoted to the samples. The samples were incubated at 4°C under constant rotation for 2 h. To enrich for direct HP1β interactors, samples were washed three times with a low-salt buffer (50 mM HEPES (pH 7.5), 140 mM NaCl, 1% Triton X-100) and once with a high-salt buffer (50 mM HEPES (pH 7.5), 500 mM NaCl, 1% Triton X-100). To reduce the detergent for subsequent protein digestion and proteomic analysis, samples were washed twice with TBS. After the last wash the supernatant was discarded carefully and the beads were resuspended in the elution buffer I (2 M Urea, 50 mM Tris–HCl (pH 7.5), 2 mM DTT and 20 μg/ml Trypsin) and incubated for 30 min at 37°C in a thermal shaker at 1100 rpm. Next, the supernatants were saved, and the beads were resuspended in 50 μl of elution buffer II (2 M urea, 50 mM Tris–HCl (pH 7.5), 40 mM CAA). After 5 min of incubation at 37°C, both supernatants were combined, and digestion was continued O/N at 25°C. The next day, 1% TFA was added to stop the digestion and peptides were cleaned-up on Stage Tipps consisting of three layers of C18 material (Empore) (40). Eluted and speedvac dried peptides were resuspended in 8 μl of A* buffer (0.1% TFA and 2% acetonitrile) and peptide concentrations were estimated by nanodrop at 280 nm.

### Full proteome sample preparation

For full proteome measurements cells were lysed in 6 M Guanidinium Chloride, 100 mM Tris–HCl pH 8.5 and freshly added 2 mM DTT by constant pipetting and subsequent boiling for 10 min at 99°C and 1700 rpm. Next, samples were quickly spun down and sonicated for in a Bioruptor Plus (30 s on/off interval, high setting). Protein concentrations were estimated by a BCA assay and meanwhile CAA was added to a final concentration of 40 mM. After a minimum incubation time of 20 min, 30 μg of each lysate was diluted in 30 μl of the lysis buffer and diluted 1:10 in the digestion buffer (25 mM Tris–HCl pH 8.5 and 10% acetonitrile). Next, trypsin and LysC were added in a 1:100 protease to protein ratio. Digestion was carried out O/N at 37°C and 1000 rpm. The next day, the samples were acidified with 1% TFA and cleaned-up on three layers of SDB-RPS material (Empore). After elution and vacuum drying, the samples were resuspended in 20 μl A* buffer and peptide concentrations were estimated by nanodrop at 280 nm.

### Enrichment of K-Gly-Gly peptides

The K-Gly-Gly enrichment was performed by using the PTMScan Ubiquitin Remnant Motif Kit (Cell Signaling Technology) according to the manufacturer's protocol.

Briefly, 1 × 10^8^ cells were lysed in the Urea lysis buffer (20 mM HEPES pH 8.0, 9 M urea, 1 mM sodium orthovanadate, 2.5 mM sodium pyrophosphate, 1 mM β-glycerophosphate.) and digested by Trypsin/LysC in an enzyme to protein ratio of 1:50 and 1:250, respectively. This step was carried out in duplicates for the *KAP1*^−/−^ and in triplicates for wt mESCs. Next, peptides were desalted using 200-mg tC18 Sep Pak Cartridges (Waters). After vacuum drying of the samples, peptides were resuspended in the IAP buffer (50 mM Mops (pH 7.2), 10 mM sodium phosphate, 50 mM NaCl) and addressed to K-Gly-Gly pulldown. Then, eluted peptides were desalted once more with C18 Stage Tips, dried with a speedvac and resuspended in 20 μl of A* buffer (0.1% TFA and 2% acetonitrile). Peptide concentrations were estimated by nanodrop at 280 nm.

### Mass spectrometry of ChIP-MS, full proteomes and K-Gly-Gly peptides

For mass spectrometry on a quadrupole Orbitrap mass spectrometer (Q Exactive HF-X, ThermoFisher Scientific), 300 ng of peptide solution per replicate was separated by nanoflow liquid chromatography on an Easy-nLC 1200 (ThermoFisher Scientific) during an increasing acetonitrile gradient for 120 min. As a column an in-house packed 50 cm column of ReproSil-Pur C18-AQ 1.9 μM resin (Dr Maisch GmbH) was used. The flow rate was constantly monitored and kept at 300 nl/min and the column oven temperature was fixed at 60°C. The injection was performed through a nanoelectrospray source. After each set of replicates, an additional washing step was scheduled. Data acquisition was performed in a data-dependent mode by selecting for the most abundant 12 peptides for MS/MS scans. The m/z range was set to 400–1650 *m*/*z*. The max. injection time was at 20 ms. The target value for the full scan MS spectra was 3  ×  10^6^ and the resolution at 60 000.

### MS data analysis

Raw MS files were first analyzed with the MaxQuant software package (version 1.6.0.7) (41). The FASTA files (reviewed and unreviewed) were obtained from Uniprot (version 2020). Contaminants were identified by the Andromeda search engine (42) with 245 entries. ‘Match between runs’ option was enabled and the false discovery rate for both peptides (minimum length of 7 amino acids) and proteins was set to 1%. Determination of the relative protein amounts followed the MaxLFQ algorithm (43), with a minimum ratio count of two peptides.

For the downstream analysis of the MaxQuant output, Perseus was used. Contaminants were filtered out, intensities were transformed to log_2_ and a two-sided Student's *t*-test with a permutation-based FDR of 0.05 and a fold change cut-off of log_2_ = 1 was applied.

## RESULTS

### HP1β plays a role in mESC differentiation

**Figure 1.**
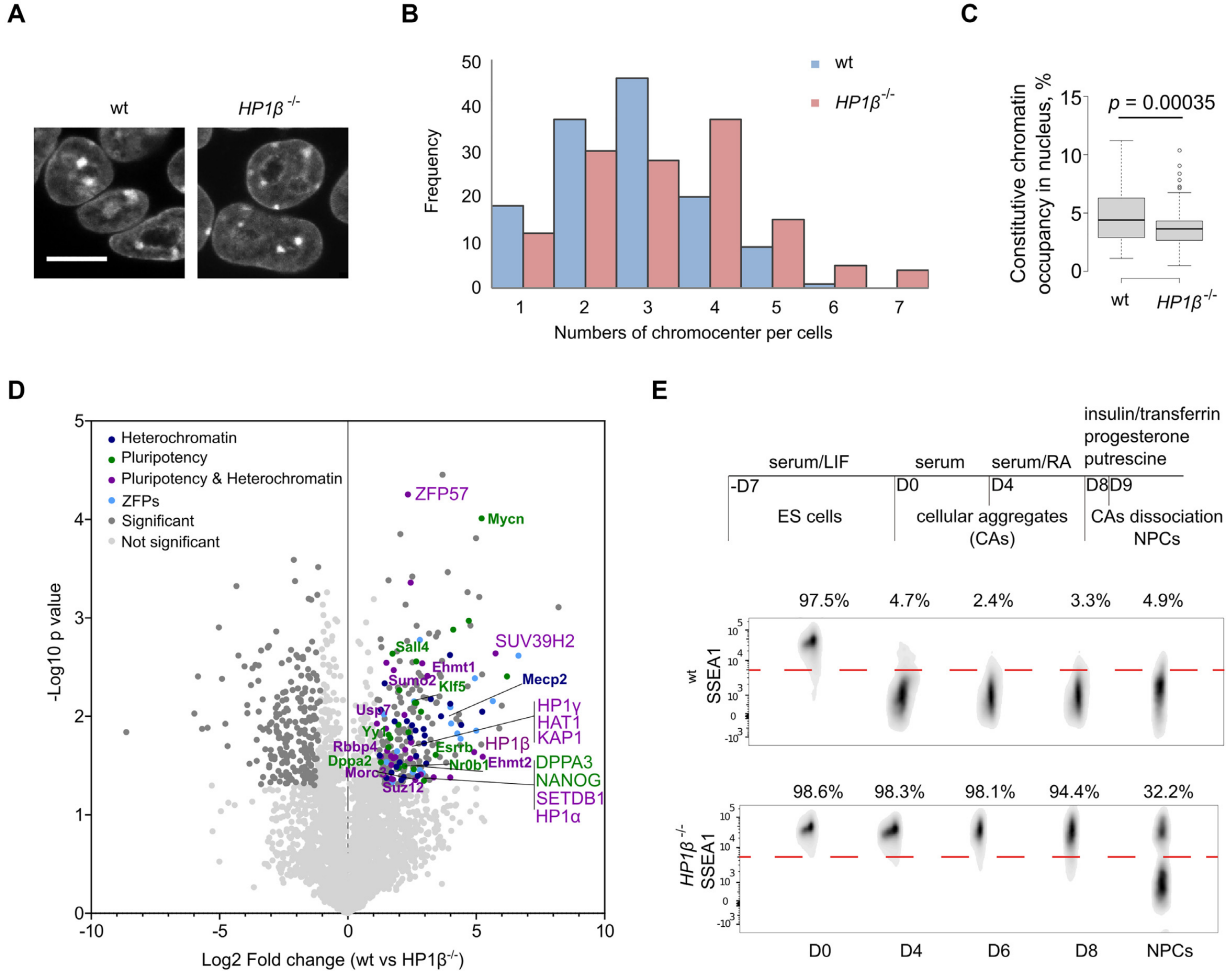
HP1β is required for neural progenitor cell (NPC) differentiation. (**A**–**C**) Depletion of HP1β leads to alterations in number and size of chromocenters. Images of mESCs stained with DAPI (A), scale bar: 10 μm. 131 nuclei for wt and *HP1β*^−/−^ cells respectively were counted and frequency (y-axis) relative to the number of chromocenters per cell (x-axis) was plotted (B). The area of chromocenters and nucleus was measured with ImageJ to calculate the relative space occupied by chromocenters within the nucleus for wt and *HP1β*^−/−^ mESCs as depicted in the box plot. Center lines show the medians; box limits indicate the 25th and 75th percentiles as determined by R software; whiskers extend 1.5× the interquartile range from the 25th and 75th percentiles, outliers are represented by dots. 109 and 102 individual cells were measured for wt and *HP1β*^−/−^, respectively. Two-sided Student's t-test was done, **** *P*< 0.001 (C). (**D**) Volcano plot from HP1β ChIP-MS in wt and *HP1β^−/−^* mESCs (*n* = 2 biological replicates). Dark gray dots: significantly enriched proteins. Blue dots: proteins involved in heterochromatin regulation. Green dots: proteins involved in pluripotency. Purple dots: proteins involved in both heterochromatin and pluripotency. Cyan dots: zinc finger proteins (ZFPs). Statistical significance determined by performing a Student's *t* test with a permutation-based FDR of 0.05 and a cutoff of <2-fold enriched proteins. (**E**) Schematic representation of the NPC differentiation strategy and more details in Materials and Methods. Cells from distinct stages of differentiation were stained with a DyLight 650-conjugated anti-SSEA-1 antibody and analyzed by FACS.

Recently we found that HP1β shows phase separation properties in the presence of H3K9me3 histones *in vitro* (11). We next investigated its function in heterochromatin organization in cells. To this end, we first inserted a multifunctional integrase (MIN) tag directly after the ATG start codon of *HP1β* (Supplementary Figure S1A) for subsequent systematic studies applying our previously described genome engineering strategy (22) (Supplementary Table S1). In a second step we used Bxb1 mediated recombination to insert a transcription termination sequence into the MIN, i.e. directly after the ATG, to generate *HP1β*^−/−^ mESCs (Supplementary Figure S1B). Immunostaining and reverse transcription quantitative PCR (RT-qPCR) showed that HP1β was completely depleted from the cells (Supplementary Figure S1C and D). DAPI staining of DNA showed alteration in chromocenter number and size in the *HP1β^−^*^/−^ compared to the wt mESCs (Figure 1A–C). *HP1β^−^*^/−^ cells exhibit an increased number of chromocenters which were on average smaller in size, indicating a reduced chromocenter clustering.

To profile the HP1β interactome, we performed chromatin immunoprecipitation coupled to mass spectrometry (ChIP-MS) of HP1β in wt and *HP1β*^−/−^ mESCs. Among the HP1β interaction partners, we found SUV39H1/2, HP1α and KAP1 (Figure 1D), all proteins involved in regulation of chromatin compartmentalization (15). We also detected several zinc finger proteins (ZFPs) and transcriptional factors involved in pluripotency regulation (highlighted in cyan and green, respectively, Figure 1D).

As chromatin reorganization is a shared feature of multiple differentiation pathways (44–46), we investigated the role of HP1β in this process. To this aim, we differentiated *wt* and *HP1β^−^*^/−^ mESCs to neural progenitor cells (NPCs, Figure 1E) (27). To monitor differentiation, we analyzed the expression of stage-specific embryonic antigen-1 (SSEA-1), a marker of pluripotent cells, at the distinct stages of NPC differentiation. Before LIF removal (stage D0) both wt and *HP1β*^−/−^ mESCs were pluripotent as evidenced by high SSEA-1 expression (Figure 1E). At D4 of the differentiation protocol less than 5% of the wt cells were SSEA-1 positive, while more than 90% of the *HP1β*^−/−^ mESCs were still SSEA-1 positive and even at NPC commitment (D9) 32.2% of the cells still expressed the SSEA-1 marker (Figure 1E). These results suggest that depletion of HP1β impairs the exit from the pluripotent state.

### HP1β is phosphorylated at serine 89 residue (HP1β-pS89) by casein kinase 2 (CK2)

**Figure 2.**
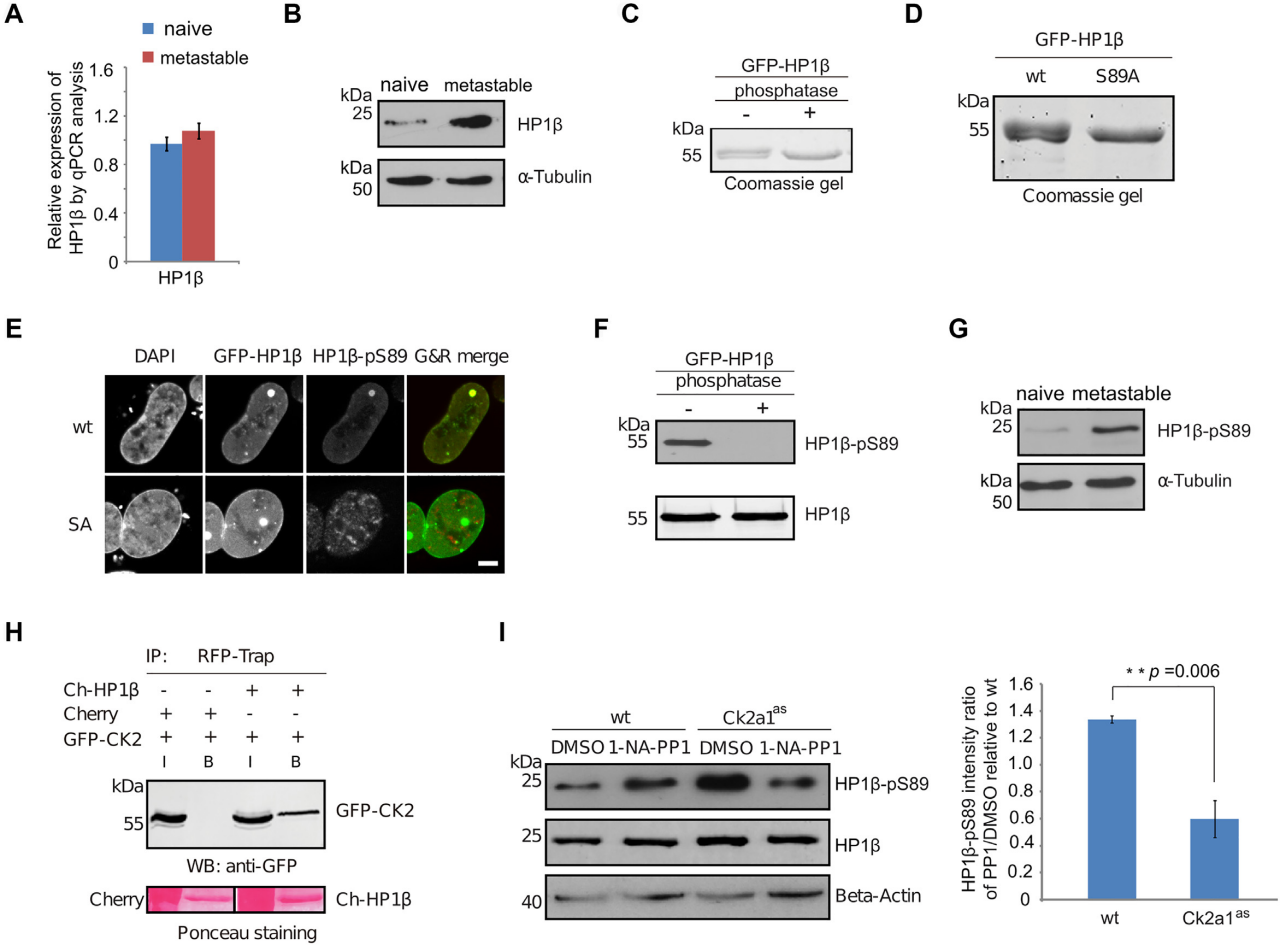
HP1β is phosphorylated at serine 89 residue. (**A**) Relative expression of HP1β in 2i/LIF (naïve) and metastable state conditions by RT-qPCR analysis. Values represent mean ± SEM from four biological replicates. (**B**) HP1β is upregulated in the metastable state condition. Total cell lysates of mESCs from naive and metastable culturing conditions were separated and visualized by anti-HP1β antibody. The anti-Tubulin blot was used as a loading control. (**C**, **D**) HP1β is highly phosphorylated on the serine 89 residue. GFP-HP1β purified from HEK293T cells was incubated with alkaline phosphatase and visualized in a coomassie stained gel (C). GFP-HP1β, wt and mutant, purified from HEK293T cells are visualized in a coomassie stained gel (D). (**E**) Characterization of a HP1β-pS89 monoclonal antibody by immunostaining. GFP-HP1β wt and mutant GFP-HP1β S89A fusion proteins were transiently expressed in BHK cells. HP1β proteins were anchored at a lac operator (*lacO*) array inserted in the genome and visible as a spot of enriched GFP fluorescence in the nucleus. Cell nuclei were stained with DAPI and HP1β proteins were visualized by the HP1β-pS89 antibody, scale bar: 5 μm. (**F**) Characterization of HP1β-pS89 monoclonal antibody by western blot. GFP-HP1β purified from HEK293T cells was incubated with alkaline phosphatase and visualized with anti-HP1β-pS89 antibody. (**G**) HP1β-pS89 is upregulated in the metastable condition. Total cell lysates of mESCs from naive and metastable culturing conditions were separated and visualized by anti-HP1β antibody. The anti-Tubulin blot was used as a loading control. (**H**) Co-immunoprecipitation shows an interaction between GFP-CK2 and Ch-HP1β. Cherry alone and cherry-tagged HP1β were immunoprecipitated from HEK293T cells co-transfected with GFP-CK2 using a RFP-Trap. Bound fractions were separated and visualized with an anti-GFP antibody and ponceau staining. (**I**) HP1β-pS89 is downregulated in the CK2a1^as^ cell line treated with 1-NA-PP1. Total cell lysates from wt and CK2a1^as^ mESCs treated with DMSO or 1-NA-PP1 were separated and visualized with anti-HP1β-pS89 and anti-HP1β antibodies. The anti-Actin blot was used as a loading control. Intensities of HP1β-pS89 were measured with ImageJ and normalized to the corresponding intensities of Actin before intensity ratios (1-NA-PP1/DMSO) were calculated. Values represent mean ± SEM of four biological replicates and the *P*-value of a two-sided Student's t-test is indicated.

To dissect the role of HP1β in pluripotency exit, we cultured mESCs with 2i/LIF (naïve) and serum/LIF (metastable state) media. In contrast to the naïve mESCs cultured with 2i/LIF, most cells in metastable state exhibit an altered transcriptional and epigenetic profile related to preimplantation epiblast cells (primed) (47,48). At the transcriptional level HP1β showed the lowest expression of all HP1 genes, with no significant changes at naïve and metastable state culturing conditions (Figure 2A and Supplementary Figure S2). However, we detected ∼2–3 fold increase of HP1β protein abundance by western blot analysis in the metastable state condition (Figure 2B).

Investigating possible posttranslational modifications of HP1β we noticed that GFP-HP1β purified from HEK293T cells migrates in coomassie stained protein gels as a double band of which the upper one disappeared upon incubation with antarctic phosphatase (Figure 2C). With mutational analyses, we mapped a phosphorylation at the serine 89 residue (Figures 2D, Supplementary Figure S3A and B). The phosphorylation of GFP-HP1β at the serine 89 residue was also detected with mESCs (Supplementary Figure S3C). To characterize the function of HP1β phosphorylation, we generated a monoclonal antibody against HP1β-pS89 (Figure 2E and F). With this antibody, we stained mouse rod photoreceptor cells, which display three distinct and spatially separated classes of chromatin, to assay for altered binding preferences of HP1β-pS89 but found a similar heterochromatin distribution as for HP1β (Supplementary Figure S3D). We observed an increase of HP1β-pS89 in the metastable condition by western blot in the absence of transcriptional changes (Figure 2G). This phosphorylation might stabilize HP1β and, thus, in the absence of transcriptional changes, contribute to increased protein levels at the transition from naive to primed state.

The serine 89 residue is located within a sequence of S/TxxE/D that is the consensus recognition motif for casein kinase 2 (CK2, Supplementary Figure S3A). As we also found a physical interaction between CK2 and HP1β (Figure 2H), we introduced a CK2a1 analog sensitive mutation (CK2a1^as^) into wt mESCs by CRISPR-Cas9 (Supplementary Figure S3E). This analog sensitive mutation allows for rapid and highly specific CK2a1 inhibition with the adenine analog 1-NA-PP1 (49), which does not affect other kinases and wt cells. Upon addition of the adenine analog, we observed a clear reduction of HP1β-pS89 level (Figure 2I). Additionally, we treated cells expressing GFP-HP1β protein with the specific CK2 inhibitor 4,5,6,7-tetrabromobenzotriazole (TBB). Analysis of the phosphorylated to unmodified HP1β ratio in a coomassie stained protein gel indicated a clear reduction with TBB treatment (Supplementary Figure S3F). These results suggest that the phosphorylation of HP1β is catalyzed by CK2.

### Phosphorylation enhances the phase separation of HP1β *in vitro*

**Figure 3.**
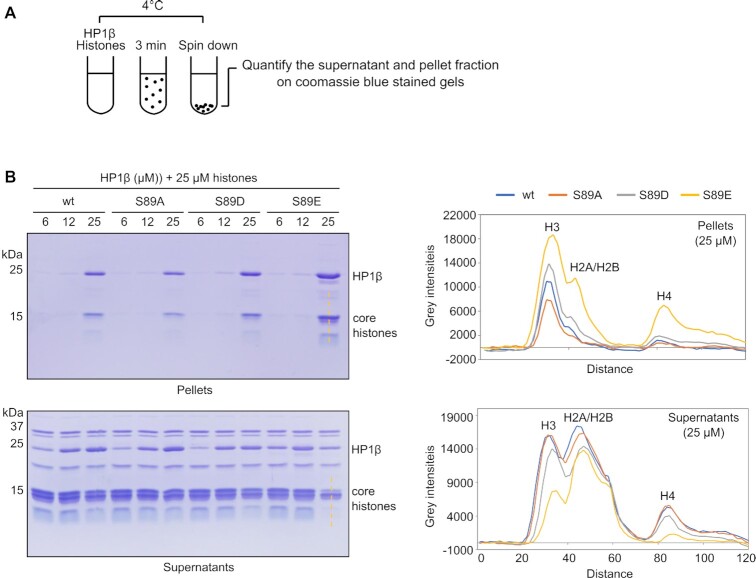
HP1β phosphorylation enhances its phase separation. (**A**) Illustration of the spin down assay to separate phase droplets from solution. (**B**) HP1β variants from 6 to 25 μM were incubated with 25 μM histones and phase-separated droplets were collected by centrifugation. Proteins in supernatants and pellets were visualized in coomassie stained gels. Line scans along the core histones in the supernatants and pellets of HP1β wt and mutant droplets at the concentration of 25 μM.

Phase separation of HP1 is involved in regulation of heterochromatin formation (8,9). We recently showed that the charge of IDR-H determines the phase separation of HP1 homologues. HP1β forms phase separated droplets in the presence of core histones *in vitro* (11). The phosphorylation of serine 89 adds additional negative charge to the IDR-H of HP1β and lowers the p*I* to 5.3. To investigate the function of HP1β phosphorylation in phase separation, we purified HP1β wt and its mutants including HP1β S89A, HP1β S89E and HP1β S89D and incubated different amounts of the HP1β proteins (from 6 to 25 μM) with 25 μM histones. We collected phase-separated droplets by centrifugation and quantified the precipitated HP1β and histones with coomassie stained gels (Figure 3A and B). In contrast to the HP1β wt and non-phosphorylatable mutant HP1β S89A, the mutants mimicking HP1β phosphorylation (HP1β S89D and HP1β S89E), were more efficient in forming phase-separated droplets at the concentration of 25 μM as more histone H3 was depleted from supernatants and enriched in the pellets (Figure 3B). These results suggest that the phosphorylation of HP1β at S89 enhances phase separation in the presence of histones, probably through weak interactions between the acidic IDR-H of HP1β and basic histones.

### HP1β-pS89 promotes mESCs exit from naïve pluripotent state

**Figure 4.**
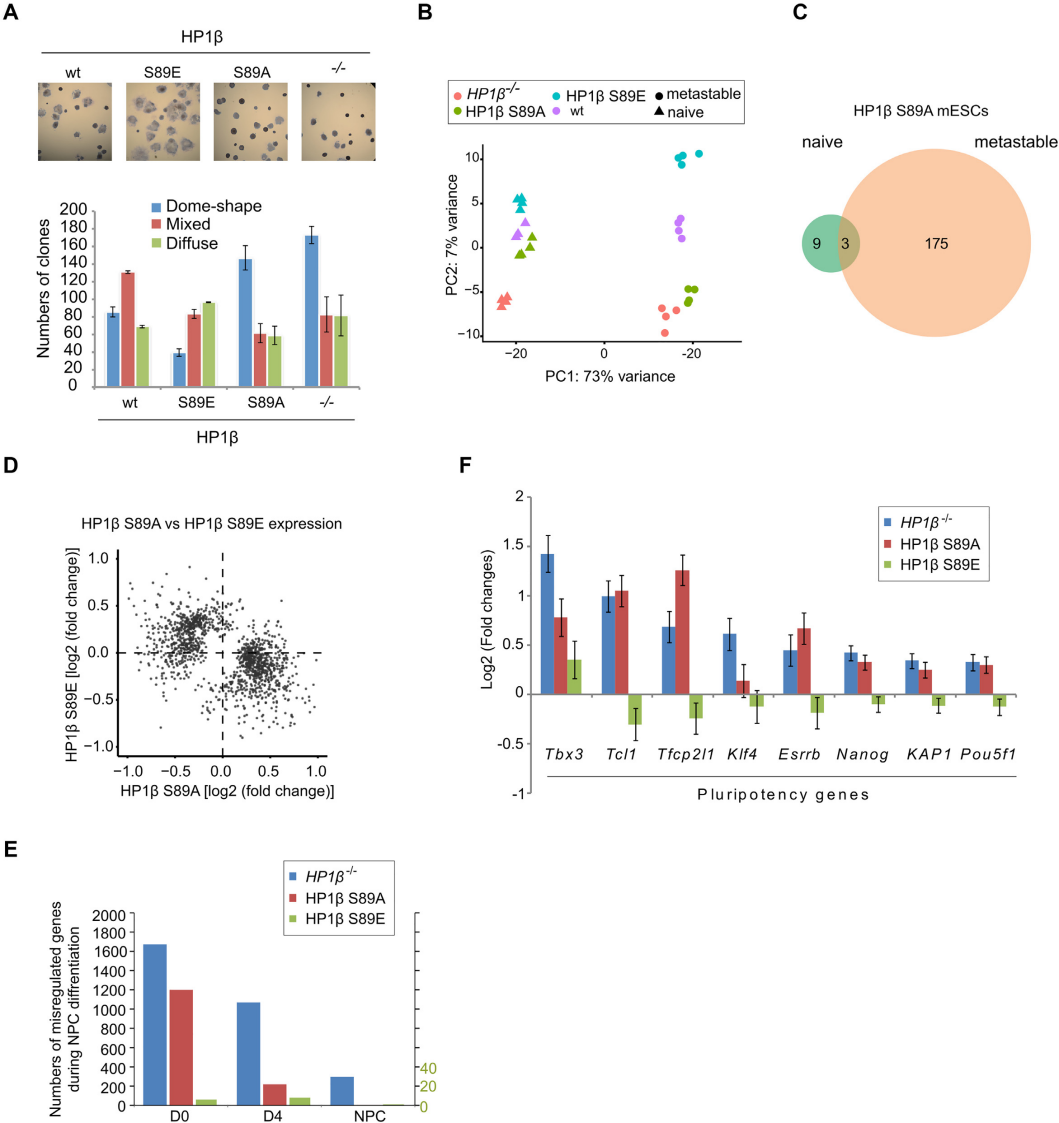
HP1β-pS89 promotes mESCs exit from naïve pluripotent state. (**A**) Representative images show alkaline phosphatase (AP) staining of wt E14 and HP1β mutant mESCs cultured in serum/LIF medium for 6 days. Numbers of dome-shape, diffuse and mixed colonies were counted, and values represent mean ± SEM from two different clones, each as a biological triplicate. (**B**) Principal component analysis (PCA) of whole transcriptome RNA-seq data from indicated cell lines in naive and metastable conditions. (**C**) Venn diagram showing dysregulated genes with fold changes >1.5 in HP1β S89A mESCs in naive and metastable conditions. (**D**) Scatter plot depicts overlapping dysregulated genes of HP1β S89A and HP1β S89E. (**E**) Bar plot showing the number of dysregulated genes from the transcriptomes of *HP1β*^−/−^, HP1β S89A and HP1β S89E mESCs at the indicated stages of NPC differentiation. (**F**) Pluripotency genes found to be dysregulated in (E) were plotted for the respective cell lines.

To investigate the function of HP1β S89 phosphorylation, we generated mESCs expressing either the non-phosphorylatable HP1β S89A or the phosphomimetic HP1β S89E (Supplementary Figure S4A and B). Western blot and immunostaining indicated that the mutant mESCs express a similar HP1β level to wt cells (Supplementary Figure S4C and D). In mESC cultures we noticed that *HP1β*^−/−^ and HP1β S89A cells formed dome-shape colonies under metastable culture condition, while wt and HP1β S89E mESCs cultures were heterogeneous with mixed dome-shape and differentiated colonies. To quantify the morphology changes, we performed colony-formation assays and observed that *HP1β*^−/−^ and HP1β S89A mESCs formed more naïve-like compact dome-shaped colonies (Figure 4A). We also observed that under metastable culture conditions *HP1β*^−/−^ and HP1β S89A mESCs maintained the lower proliferation rate typical for the naïve state, while HP1β wt and HP1β S89E ESCs more than doubled (Supplementary Figure S4E). The fact that *HP1β*^−/−^ and HP1β S89A mESCs continue to resemble naïve ESCs in terms of morphology and proliferation under metastable culture conditions suggests a possible defect in the exit from pluripotency.

To further investigate the role of HP1β we performed RNA-seq analysis of wt E14 and mutant mESCs, including *HP1β*^−/−^, HP1β S89A and HP1β S89E cells cultured in both naive and metastable conditions. Principal component analysis (PCA) of transcriptomes revealed a significant separation between these two culture conditions reflecting the extensive changes in gene expression at the exit from pluripotency (Figure 4B). While the phosphorylation status of HP1β did not seem to matter in the naïve state, extensive differences were observed under metastable culture conditions. mESCs with the non-phosphorylatable HP1β S89A were widely separated from phosphomimetic HP1β S89E cells and closely resembled *HP1β*^−/−^ cells in the PCA (Figure 4B).

Thus, the phosphorylation mutation (HP1β S89A) significantly affected the expression of 178 genes (fold change >1.5) in metastable state but only 12 genes in naive state (Figure 4C and Supplementary Table S2). Interestingly, HP1β S89A and HP1β S89E affected gene expression in opposing ways (Figure 4D). Gene Ontology (GO) enrichment analyses of biological processes showed that axis specification and cell differentiation were observed in both *HP1β*^−/−^ and HP1β S89A (Supplementary Figure S5A). Also, we found that the dysregulated genes in *HP1β*^−/−^ and HP1β S89A cells overlapped with the pluripotency cell fate (PCF) genes identified previously (50) (Supplementary Figure S5B).

Next, we further differentiated wt E14 and HP1β mutant mESCs to NPCs and analyzed their transcriptomes at distinct stages of differentiation (Supplementary Table S3). Notably, the transcriptomes of *HP1β*^−/−^ and HP1β S89A cells showed dramatic changes in contrast to HP1β S89E, especially at the D0 of differentiation (Figure 4E). In agreement with the colony formation assay (Figure 4A), we found pluripotency genes such as *Tfcp2l1*, *Esrrb* and *Nanog* marker for naïve pluripotency state (51,52), upregulated in both *HP1β*^−/−^ and HP1β S89A cells and slightly downregulated in HP1β S89E cells (Figure 4F and Supplementary Table S3). Collectively, these morphology, proliferation and gene expression data indicate that phosphorylation of HP1β at S89 is necessary for mESCs exit from the naïve pluripotent state.

### HP1β-pS89 binds and sequesters KAP1 in heterochromatin compartments

**Figure 5.**
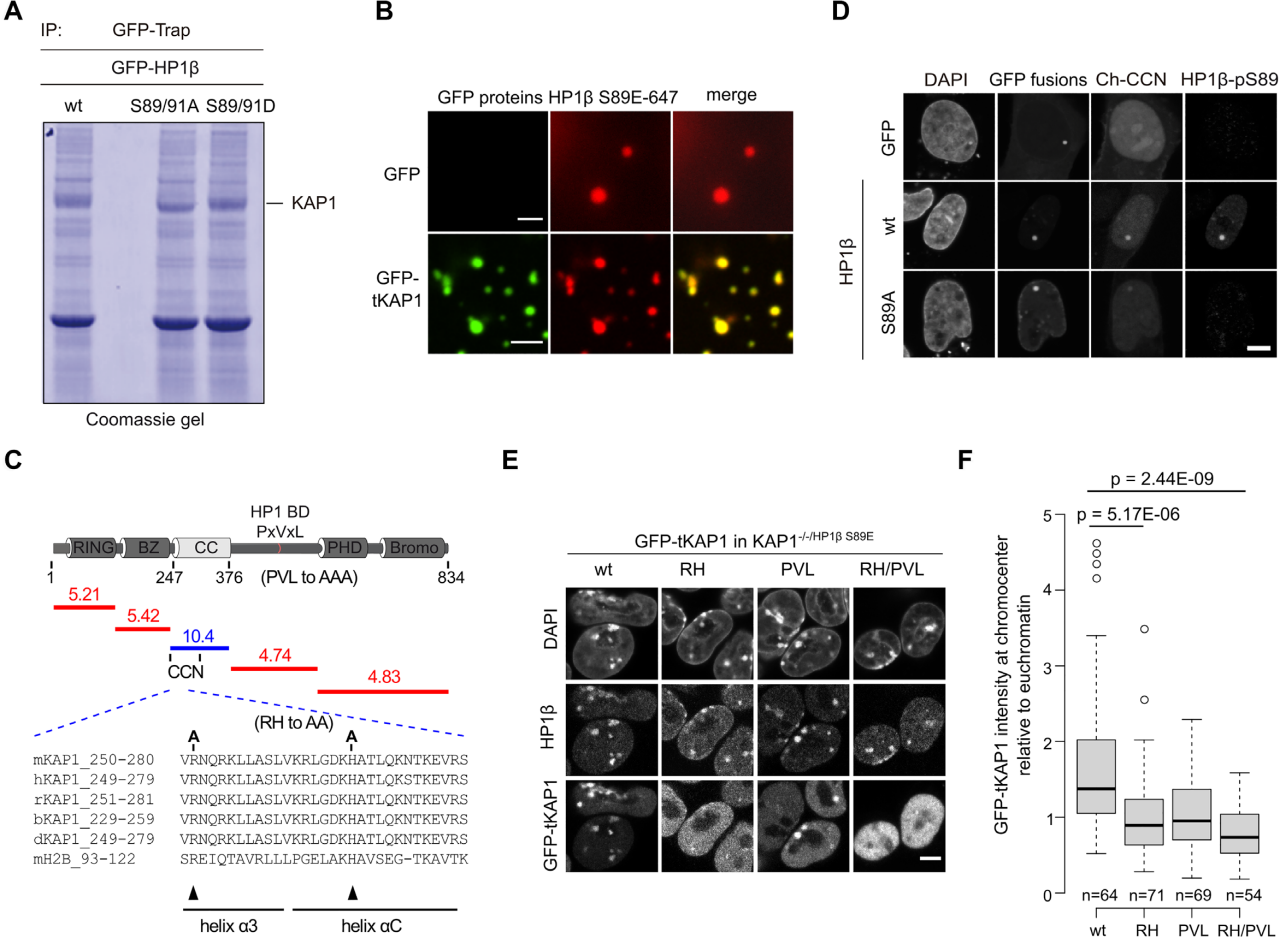
HP1β-pS89 interacts and recruits KAP1 to heterochromatin. (**A**) GFP-HP1β proteins immunoprecipitated using a GFP-Trap from HEK293T cells were separated and visualized by coomassie stained gels. A band showing more in GFP-HP1β wt and GFP-HP1β S89D, but less GFP-HP1β S89A, was cutted and sequenced by MS. (**B**) KAP1 is enriched in HP1β S89E phase-separated droplets *in vitro*. GFP and GFP-tKAP1 purified from HEK293T cells were incubated with 25 μM of HP1β S89E and histones in a buffer of 20 mM HEPES pH 7.2, 75 mM KCl and 1 mM DTT. 30 nM of HP1β S89E labeled with a NT-647 dye was added and phase-separated droplets were imaged using a 63x objective on a DeltaVision Personal Microscopy at 63 ×, scale bar: 5 μm. (**C**) Schematic illustration of KAP1 domains and their respective pI values. RING: really interesting new gene, BZ: B-box zinc finger, CC: Coiled-Coil, HP1 BD PxVxL: HP1 binding motif, PHD: plant homeodomains and Bromo domains. The N-terminus of CC (CCN) comprises a sequence (aa 250–280) that shares similarity with mouse histone H2B (aa 93–122). Conserved amino acids are highlighted in blue. (**D**) The CCN interacts with HP1β-pS89. To use the fluorescence three hybrid assay (F3H) (Herce *et al.*, 2013; Rothbauer *et al.*, 2008), GFP and GFP-HP1β fusion proteins as well as Ch-CCN were transiently expressed in BHK cells. GFP and GFP-HP1β proteins are anchored at a lac operator (*lacO*) array inserted in the BHK genome, thereby leading to a spot of enriched GFP fluorescence within the nucleus. While GFP-HP1β showed accumulation of Ch-CCN at the lacO spot, no or only weak interactions were detected for GFP and GFP-HP1β-SA, respectively. HP1β-pS89 was visualized with an anti-HP1β-pS89 antibody and nuclei were stained with DAPI, scale bar: 5 μm. (**E**) Images show *KAP1^−/−^*^/HP1β S89E^ mESCs stably expressing either GFP-KAP1 wt or RH/PVL single or double mutation stained with an anti-HP1β antibody and DAPI, scale bar: 5 μm. (**F**) Quantification of chromocenter enrichment of GFP-tKAP1 wt and its mutations. GFP intensities in the chromocenters and euchromatic regions were measured with ImageJ and their ratio was calculated. Center lines depict the median; box limits indicate the 25th and 75th percentiles as determined by R software; whiskers extend 1.5x the interquartile range from the 25th and 75th percentiles; outliers are represented by circles. Individual chromocenters were analyzed (*n* = 64, 71, 69, 54 for GFP-tKAP1 wt, RH, PVL and RH/PVL, respectively). *P* values of a two-sided Student's *t*-test are indicated.

To investigate how S89 phosphorylation affects the HP1β interactome, we generated HEK293T cell lines stably expressing either GFP-HP1β wt or the non-phosphorylatable mutant GFP-HP1β S89A or the phosphomimetic GFP-HP1β S89D. Since the serine 91 residue is close to serine 89 and was identified as an alternative phosphorylation site previously (53) (Supplementary Figure S6), it was also mutated to alanine in this assay. Interacting proteins were co-immunoprecipitated from cell extracts and compared by coomassie stained gels. A protein band specific for the phosphomimetic GFP-HP1β S89D was cut out and identified by MS analysis, as KAP1 (Figure 5A and Supplementary Figure S7). To test whether KAP1, as an interacting protein, is recruited by HP1β, we added truncated GFP-tKAP1 (aa 114–834) and found that it was specifically enriched in HP1β S89E phase-separated droplets *in vitro* (Figure 5B). Previously, the PxVxL motif of KAP1 (also known as HP1 box) was shown to bind the HP1 CSD (54,55). However, the observation that phosphorylation of S89 in the IDR-H of HP1β enhances the interaction with KAP1 (Supplementary Figure S7), suggests that KAP1 comprises a second site, besides the PxVxL motif, that specifically recognizes the phosphorylated HP1β.

In the amino acid sequence of mouse KAP1 the region from aa 247–376, known as coiled-coiled (CC) domain, stands out by its extreme basicity reaching a pI of 10.4 (Figure 5C) which makes it a good candidate to bind the acidic IDR-H of HP1β and to discriminate the phosphorylation at S89. A closer inspection of this CC domain revealed a striking similarity of the N-terminal aa 250–324 (CCN) with the C-terminus of H2B (helix α3 and helix αC) (Figure 5C). With a fluorescence three hybrid protein-protein interaction (F3H) assay (25,56), we could show that this CCN subdomain of KAP1 specifically binds HP1β-pS89 but not the non-phosphorylatable HP1β S89A (Figure 5D). These results fit well with the recent observation that sites within the IDR-H of HP1 interact with core histones (13).

**Figure 6.**
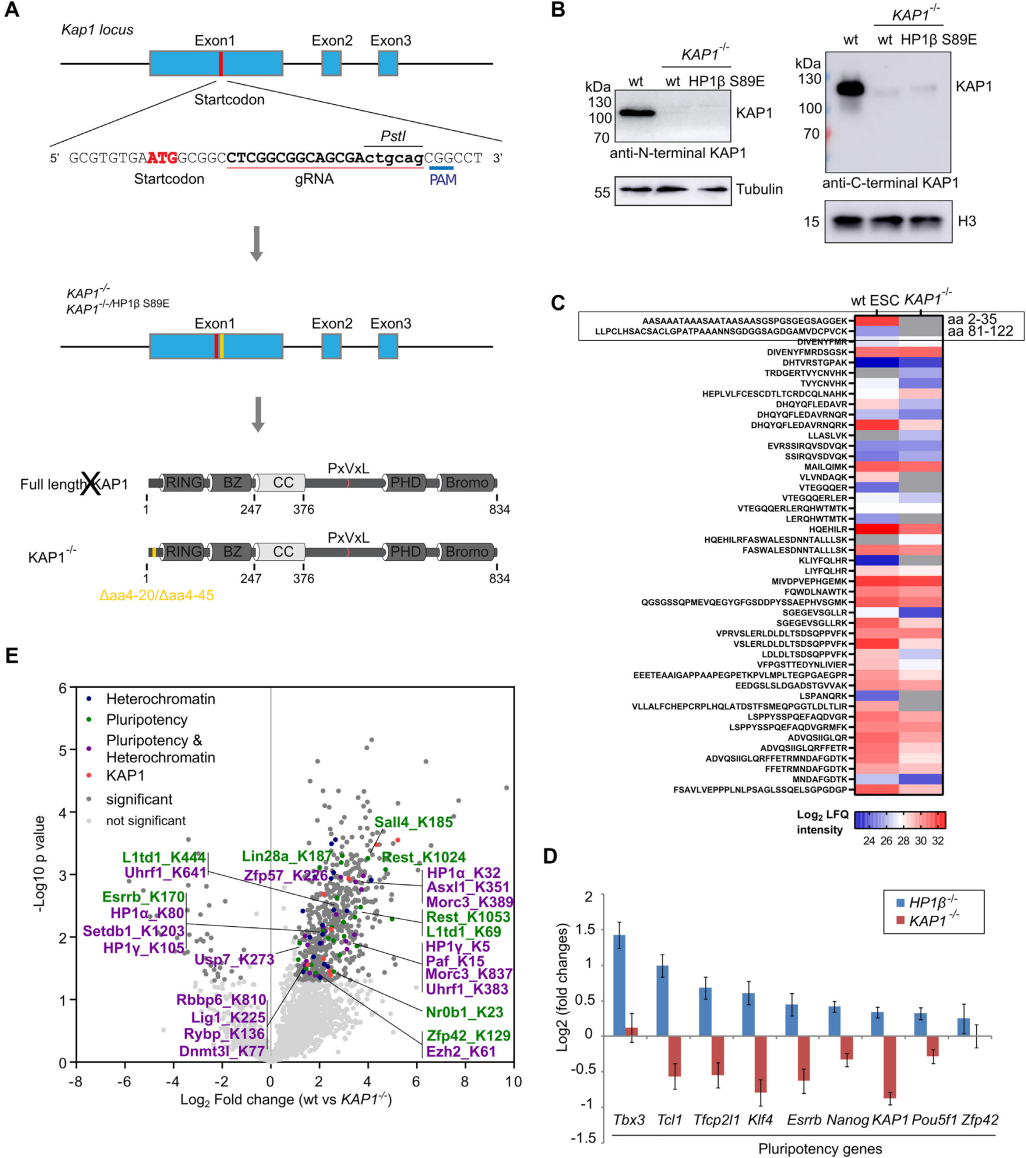
KAP1 relies on its ubiquitination activity to regulate pluripotency. (**A**) Schematic representation shows the CRISPR/Cas9 gene editing strategy used to generate *KAP1*^−/−^ mESCs. gRNA target sequence and restriction enzyme recognition sites for screening are shown. (**B**) Western blot analysis of KAP1 protein levels in wt and *KAP1^−/−^* mESCs using antibodies against N- (left) and C-terminus (right) of KAP1. The tubulin and H3 blots were used as loading controls. (**C**) Mass spectrometry analyses of KAP1 expression in wt and *KAP1^−/−^* mESCs. (**D**) Volcano plot from diGly pulldowns in wt (*n* = 3 biological replicates) and *KAP1^−/−^* mESCs (*n* = 2 technical replicates). Dark gray dots: significantly enriched proteins. Blue dots: proteins involved in heterochromatin regulation. Green dots: proteins involved in pluripotency. Purple dots: proteins involved in both heterochromatin and pluripotency. Red dots: KAP1. Statistical significance determined by performing a Student's *t* test with a permutation-based FDR of 0.05 and an additional constant S0 = 1. (**E**) Plot of dysregulated pluripotency genes in the transcriptomes of *HP1β*^−/−^ and *KAP1^−/−^* mESCs. Dark gray dots: significantly enriched proteins. Blue dots: proteins involved in heterochromatin regulation. Green dots: proteins involved in pluripotency. Purple dots: proteins involved in both heterochromatin and pluripotency. Red dots: KAP1 peptides.

To test the relative contribution of both, CCN and PxVxL, domains toward HP1β binding, we generated first an mESC line lacking KAP1 and expressing the phosphomimetic HP1β S89E (*KAP1^−/−^*^/HP1β S89E^ cell line; Figure 6A). We, then, tested complementation with GFP-tKAP1 fusion proteins with mutated CCN (RH) and/or PxVxL (PVL) domains (Figure 5E) for enrichment at heterochromatic chromocenters. The comparison with wt GFP-tKAP1 shows that both single mutations reduce the enrichment at DAPI stained chromocenters and the double mutation (RH/PVL) mostly abolished KAP1 localization at chromocenters (Figure 5E and F). These results indicate that KAP1 CCN and PxVxL both contribute to HP1β binding and enrichment at chromocenters, whereby the CCN subdomain at the same time recognizes the S89 phosphorylation.

### KAP1 contributes to pluripotency maintenance

As HP1β-pS89 regulates pluripotency exit and specifically interacts with KAP1, we further analyzed *KAP1*^−/−^ mESCs. These cells were generated using a gRNA that targets the site after the first start codon (Figure 6A). PCR followed by sanger sequencing showed a 51 or 126 bp deletion within exon 1 of the KAP1 locus (Supplementary Figure S8A and B). Consistently, we did not detect KAP1 with an antibody against the N-terminus (aa 1–50) but observed faint shorter KAP1 bands with an antibody against the C-terminus of KAP1 (Figure 6B). Further analyses using mass spectrometry indicated that KAP1 protein, lacking the first N-terminal region, was present (Figure 6C). We only detected N-terminal peptides of KAP1 from wt ESCs (Figure 6C). In view of the very low level of KAP1 protein in the mutant cells (Figure 6B), they can be used as *KAP1*^−/−^ mESCs. We next performed RNA-seq analysis of *KAP1*^−/−^ mESCs and analyzed the GO term enrichment of upregulated and downregulated genes to biological processes (Supplementary Figure S9A and B). We observed cell differentiation in the GO terms of downregulated genes that is also found in both *HP1β*^−/−^ and HP1β S89A. Among the misregulated genes in *KAP1^−/−^* mESCs (Supplementary Table S4), in particular naïve pluripotency genes, such as *Tfcp2l1*, *Tcl1, Esrrb* and *Nanog*, were downregulated, which is consistent with previous studies showing that KAP1 derepresses pluripotency genes (57). Interestingly, *KAP1^−/−^* mESCs show the opposite effect on gene expression as *HP1β*^−/−^ cells (Figure 6D) and resemble mESCs with phosphomimetic HP1β S89E (Figure 4F).

To investigate the mechanism of KAP1 in pluripotency maintenance, we further identified its ubiquitin targets by comparing wt and *KAP1^−/−^* cells by performing diGly pulldowns and mass spectrometry analyses as KAP1 has ubiquitin E3 ligase activity (58). Among the ubiquitin targets identified, we found the proteins that regulate heterochromatin, for example Setdb1, ZFP57, MORC3 and HP1, and several (naive) transcription factors, such as Sall4 and Esrrb (Figure 6E). These results suggest that KAP1 ubiquitinates heterochromatin regulators or transcription factors to regulate pluripotency.

### Sequestration of KAP1 in heterochromatin by HP1β-pS89 promotes pluripotency exit

**Figure 7.**
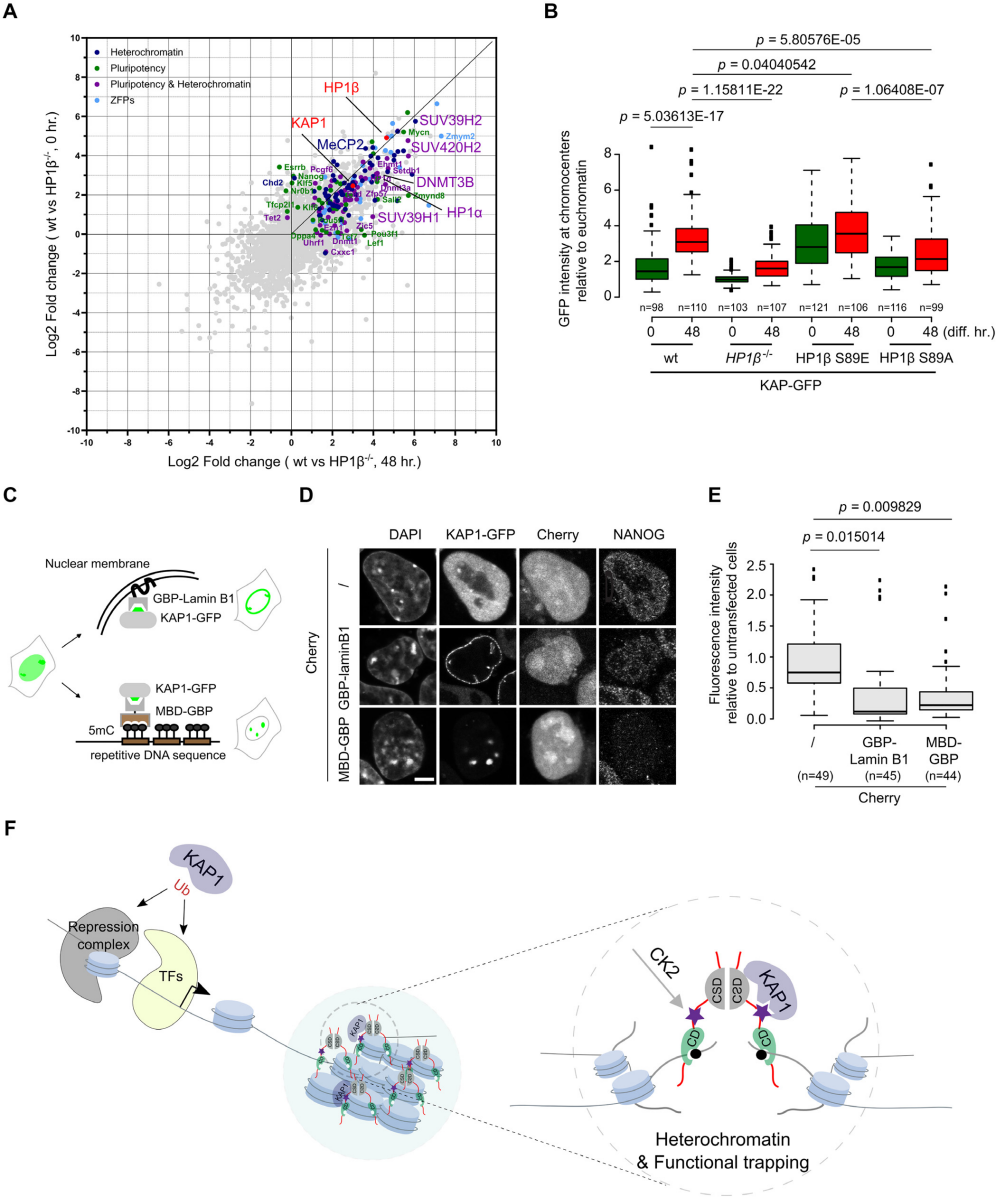
HP1β-pS89 sequesters KAP1 into heterochromatin to promote mESCs exit from pluripotency. (**A**) Comparison of the HP1β ChIP-MS under naive (0 h) and epiblast states (48 h). (**B**) KAP1 is recruited to chromocenters by HP1β-pS89 during pluripotency exit. Box plot depicts the intensity of KAP1-GFP at chromocenters relative to the signal at euchromatic regions in GFP knockin cell lines at the naïve (0h) and epiblast (48 h) state, respectively. Center lines show the medians; box limits indicate the 25th and 75th percentiles as determined by R software; whiskers extend 1.5x the interquartile range from the 25th and 75th percentiles, outliers are represented by dots. The number of chromocenters (n) analyzed for each sample is indicated. *P* values from a two-sided Student's t-test are indicated. (**C**) Schematic representation of tethering KAP1-GFP to the nuclear envelope and chromocenters by using GBP-Lamin B1 and MBD-GBP, respectively. (**D**) Representative images of *HP1β*^−/−^ cells ectopically expressing Cherry in combination with GBP-Lamin B1 or MBD-GBP stained with NANOG and DAPI, scale bar: 5 μm. (**E**) Box plots depict relative levels of the pluripotency protein NANOG for cells showing nuclear envelope and chromocenter tethering of GFP-tagged KAP1. Fluorescence intensities in nuclei were measured with ImageJ and normalized to the signals for untransfected cells. Center lines show the medians; box limits indicate the 25th and 75th percentiles as determined by R software; whiskers extend 1.5× the interquartile range from the 25th and 75th percentiles, outliers are represented by dots. The number of cells (*n*) analyzed for each sample is indicated. Two-sided Student's *t*-test was performed, and p values are indicated. (**F**) HP1β dimerizes and binds H3K9me3 clustering chromatin to form heterochromatin compartments. In response to pluripotency exit, HP1β is phosphorylated at serine 89 residue (HP1β-pS89) by CK2, thereby sequestering KAP1 into heterochromatin compartments. KAP1 relies on its ubiquitination/sumoylation activity to regulate pluripotency. The sequestration of KAP1 leads to downregulation of pluripotency genes allowing mESCs to exit pluripotency.

We used ActivinA/FGF to induce mESCs transition from naïve (0 h) to epiblast state (48 hr.) (59) and analyzed the interactome of HP1β at these two states by ChIP-MS. We observed a stronger interaction of HP1β with KAP1 and also with other heterochromatin regulators such as SUV39H1, SUV420H2 and HP1α at the epiblast state (Figure 7A). Co-immunoprecipitation using HP1β-pS89 antibodies showed that HP1β-pS89 interacts with KAP1 at the metastable state as compared with the naïve state (Supplementary Figure S10A). We hypothesized that HP1β-pS89 binds and sequesters KAP1 in the heterochromatin compartment causing *de facto* its functional depletion. To test this hypothesis, we knocked GFP into the *KAP1* locus to create a C-terminal fusion gene product (Supplementary Figure S10B) and monitored enrichment of KAP1 at chromocenters during pluripotency exit. We observed increasing chromocenter enrichment of KAP1 in wt mESCs during differentiation to epiblast state (Figure 7B). HP1β S89E showed efficient sequestration of KAP1 at chromocenters in both naïve and epiblast states in contrast to HP1β S89A (Figure 7B). These results suggest that the displacement of KAP1 to chromocenters is HP1β-pS89 dependent.

To synthetically mimic this sequestration, we expressed GFP binding nanobodies (GBP) fused with either a methyl-cytosine binding domain (MBD) to tether KAP1-GFP to chromocenters (MBD-GBP) (25,45) or Lamin B1 for tethering to the nuclear membrane (56) (Figure 7C). The sequestration of KAP1-GFP at nuclear envelope and chromocenters was monitored by fluorescence microscopy and correlated with decreased levels of the pluripotency protein NANOG (Figure 7D and E). These results support our hypothesis that KAP1 sequestration at chromocenters by HP1β-pS89 causes a functional depletion and a down-regulation of pluripotency genes.

Altogether, our results show that phosphorylation of HP1β at S89 generates a specific binding site for KAP1 and thereby captures this essential regulator of pluripotency (Figure 7F).

## DISCUSSION

HP1 proteins bind H3K9me3 and regulate chromatin organization during cell differentiation. We found that the *HP1β^−/−^* mESCs are defective in NPC differentiation. This result is consistent with a previous finding showing an impaired neuronal precursor differentiation in mouse brain (17). We found that the pluripotency exit depends on a phosphorylation of HP1β at the serine 89 residue (HP1β-pS89), as we observed similar alterations in *HP1β*^−/−^ and HP1β S89A cells at the D0 of NPC differentiation. However, only a few genes in HP1β S89A ESCs show altered expression at the NPC stage. These results suggest that HP1β-pS89 contributes to the pluripotency exit, but it is not required for the late stage of NPC differentiation.

With mutation analyses, we identified that the HP1β-pS89 is catalyzed by CK2 in cells, which is in line with *in vitro* phosphorylation assay following mass spectrometry analyses (60,61). This phosphorylation generates a specific binding site for KAP1 that provides a link to pluripotency as KAP1 has been identified as an essential factor that represses differentiation-inducible and derepresses pluripotency-associated genes (57,62–65). Consistent with this observation we found that deletion of KAP1 causes a downregulation of pluripotency genes. We identified ubiquitin targets of KAP1, such as MORC3 and HP1. Their ubiquitination may release these regulators from the promoter region that facilitates the expression of pluripotency genes. The key role of HP1β-pS89 phosphorylation in controlling this interaction with KAP1 and the exit from naive pluripotency becomes apparent from the opposite phenotypes of mESC lines with specific phosphorylation mutations. While the phospho-mimicking HP1β S89E promoted the exit from naive pluripotency, the non-phosphorylatable mutant HP1β S89A impairs this transition.

We also found that the binding of KAP1 requires the phosphorylation of HP1β at S89 in the IDR-H. In addition to the known PxVxL HP1 binding motif, which had been reported to be essential for early development (66) we identified the N-terminal part of the coiled-coil domain (CCN) of KAP1 as a second binding domain that discriminates the phosphorylation state of HP1β. Furthermore, we found that the binding of KAP1 to phosphorylated HP1β at heterochromatic chromocenters causes a depletion of free KAP1 in the nucleoplasm. We reproduced this KAP1 depletion by fusing KAP1 with GFP and captured the fusion protein at chromocenters and at the nuclear lamina with a GFP binding nanobody (GBP) fused to a methylcytosine binding domain (MBD) and lamin B, respectively. This synthetic capture caused a depletion of available KAP1-GFP and a concomitant downregulation of the NANOG pluripotency factor.

The naïve, formative and primed pluripotency states of stem cells are characterized and maintained by distinct transcriptional networks (48,50–52,59,67–69). We used 2i/LIF and serum/LIF to maintain mESCs at the naïve and metastable states, respectively. As most mESCs in metastable state exhibit an altered transcriptional and epigenetic profile relative to preimplantation epiblast cells (primed), we analyzed the cells from these two culture conditions to investigate the naïve pluripotency exit. Restricting the nuclear localization of one of these factors may destabilize the pluripotency network as was shown for the bHLH transcription factor Tfe3 (70). Our results suggest that the binding to HP1β-pS89 in chromocenters restricts the nuclear availability of KAP1 and thereby impairs the expression of pluripotency genes and promotes the exit from pluripotency.

Phase separation has been described as a novel mechanism to locally gather and enrich factors to activate genes and to enhance transcription (71–73). Our results now suggest an opposite mechanism to negatively regulate transcription. The phosphorylation of HP1β at chromocenters creates a specific binding site for the transcription regulator KAP1. This capture of an essential regulator of pluripotency genes promotes the exit from pluripotency. In addition, a previous publication suggests that the capture of KAP1 could enhance the phase separation of HP1β/nucleosomes and heterochromatin organization (15). These results also outline a new function of heterochromatin as a subnuclear compartment to capture regulatory factors and thereby remotely control gene activation and transcription at distant parts of the genome representing a novel form of remote control of transcriptional regulation.

## DATA AVAILABILITY

Sequencing data reported in this paper are available at ArrayExpress (EMBL-EBI) under accessions ‘E-MTAB-8329’ (RNA-seq).

The raw mass spectrometry proteomics data have been deposited at the ProteomeXchange Consortium via the PRIDE partner repository with the dataset identifier ‘PXD025053’.

The flow cytometry data have been deposited to FlowRepository (https://flowrepository.org/) with repository ID: FR-FCM-Z3MZ.

Supplementary Table S2 contains the list of differentially expressed genes of HP1β S89A and S89E cells at naive and metastable conditions, related to Figure 4C and D. Supplementary Table S3 contains the list of differentially expressed genes in *HP1β^−/−^*, HP1β S89A and S89E cells during NPC differentiation, related to Figure 4E and F. Supplementary Table S4 contains the list of differentially expressed genes of *KAP1*^−/−^ cells at the metastable condition, related to Figure 6D.

## Supplementary Material

gkab548_Supplemental_Files
