# Supplementary material for: Phosphorylation of the HP1β hinge region sequesters KAP1 in heterochromatin and promotes the exit from naïve pluripotency
Source: Nucleic Acids Res. 2021 Jul 2;49(13):7406–23. doi: 10.1093/nar/gkab548 (PMC8287961; doi:10.1093/nar/gkab548)
Supplement: gkab548_Supplemental_Files [file gkab548_supplemental_files.zip › Supplementary information_revised.pdf]

Supplementary information

Phosphorylation of the HP1β hinge region sequesters KAP1 in heterochromatin and promotes the exit from naïve pluripotency

Weihua Qin[1*], Enes Ugur[1,2], Christopher B. Mulholland[1], Sebastian Bultmann[1], Irina Solovei[1], Miha Modic[3], Martha Smets[1], Michael Wierer[2], Ignasi Forné[4], Axel Imhof[4], M. Cristina Cardoso[5], Heinrich Leonhardt[1*]

Supplementary information includes 10 supplementary Figures and 4 supplementary Tables.

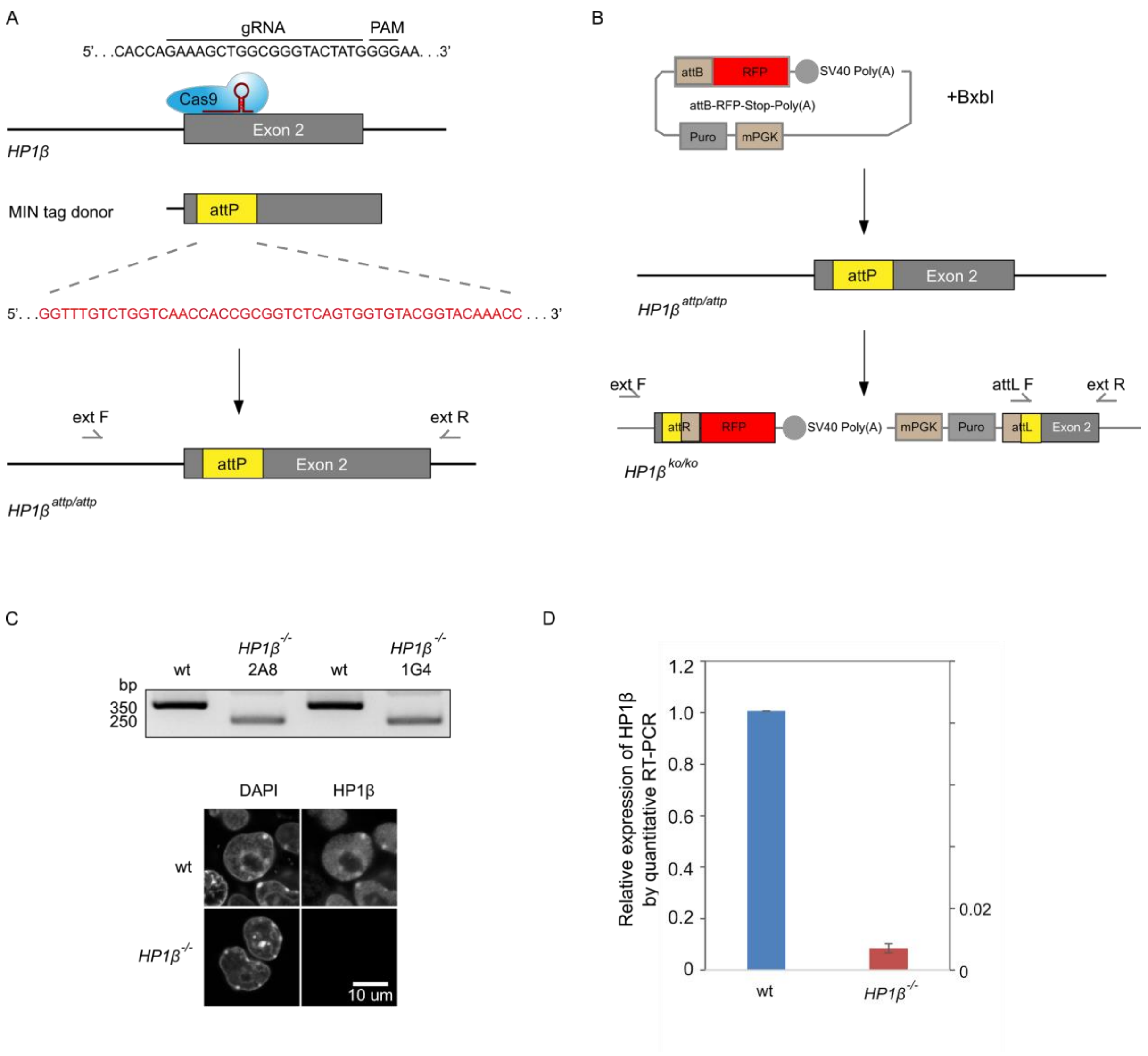

Figure S1. Generation of *HP1β*$^{-/-}$ mESC line.
(A) Schematic representations show the CRISPR/Cas9 gene editing strategy used to generate MIN tagged HP1β mESCs. The donor harbors the MIN tag sequence (attP) and homology arms to the genomic sequence 5' and 3' of the translational start site. (B) Schematic representation shows the strategy to generate *HP1β*$^{-/-}$ mESC lines with Bxb1 mediated recombination. (C) Gel electrophoresis of the multiplex PCR for wt and *HP1β*$^{-/-}$ mESCs with primers as indicated. Additional confirmation of knockouts via immunofluorescence staining using an anti-HP1β antibody. Cell nuclei were stained with DAPI. Scale bar: 10 µm. (D) Relative expression of HP1β in wt and *HP1β*$^{-/-}$ cells by RT-qPCR analysis. Values represent mean ± SEM from three biological replicates.

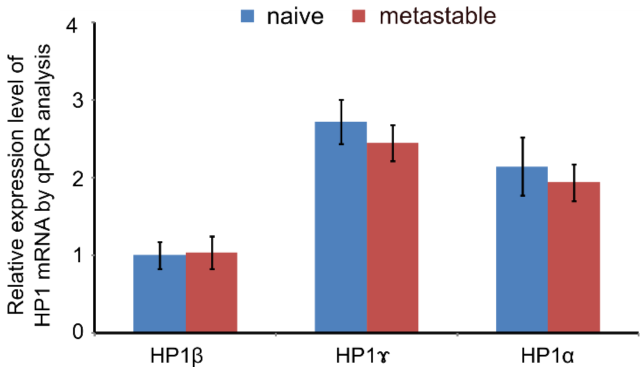

Figure S2. Expression levels of HP1 homologues.
Relative expression of HP1 homologues in 2i/LIF and metastable culturing conditions by RT-qPCR analysis. Values represent mean ± SEM from three biological replicates.

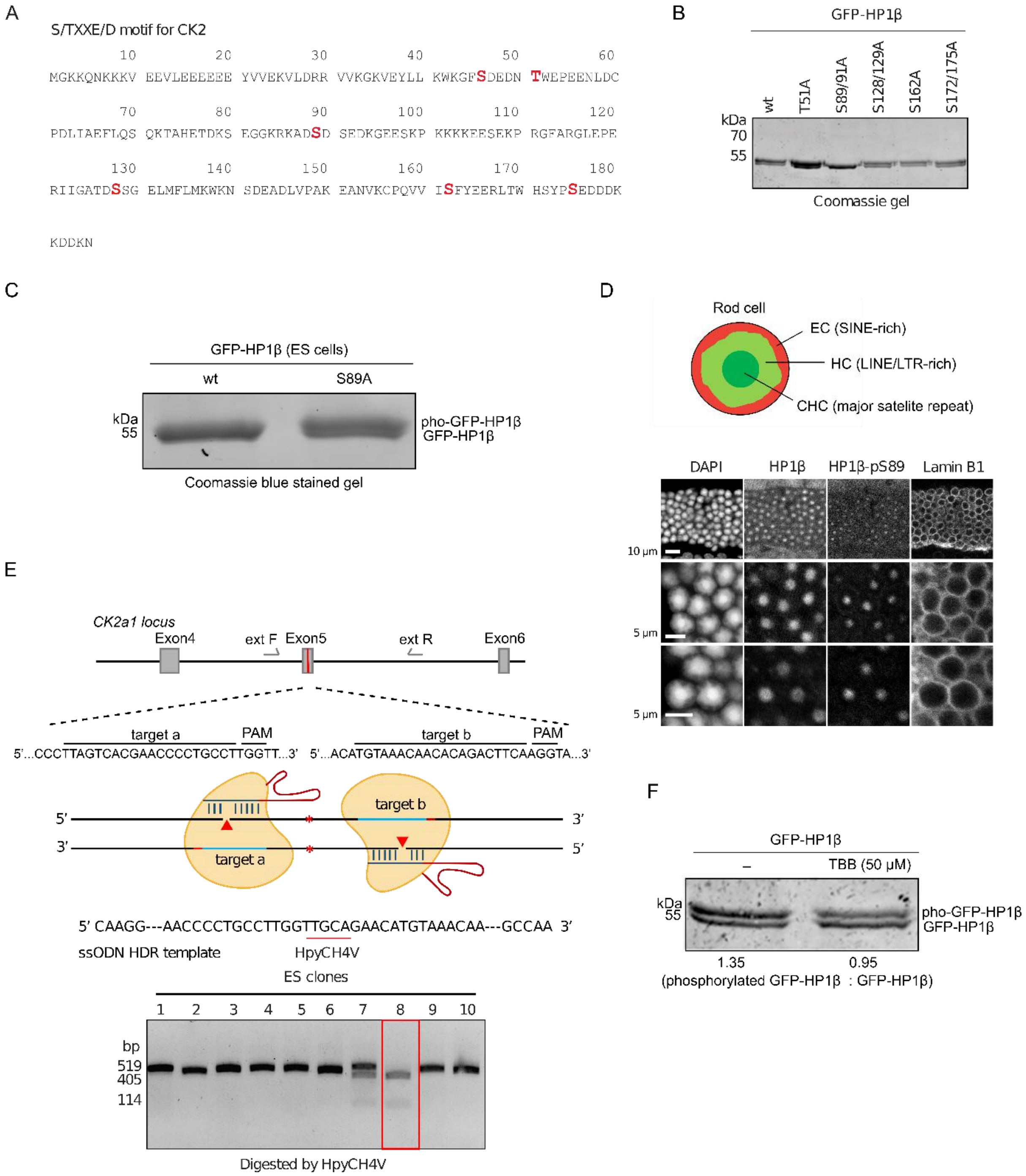

Figure S3. Characterization of HP1β-pS89.
(A) HP1β comprises several S/TxxE/D sites that represent the consensus sequence of CK2. Potentially phosphorylated serine and threonine residues are highlighted in red. (B) Phosphorylation of HP1β is mapped to the serine 89 residue by mutation analysis. GFP-HP1β wt and its mutants were purified from HEK293T cells and visualized with coomassie stained gels. (C) HP1β is highly phosphorylated on serine 89 residue. GFP-HP1β wt and mutant purified from ES cells was visualized in a coomassie stained gel. (D) Schematic representation of a mouse rod cell nucleus, EC: euchromatin, HC: heterochromatin, CHC: chromocenters. Cryosection of adult mouse retina was stained with HP1β, HP1β-pS89, lamin B1 antibodies and DAPI. Rod cells in the retina are highlighted and scale bars sizes are indicated. (E) Schematic representation shows the CRISPR/Cas9 gene editing strategy used to generate a CK2a1^{as} mESC line. The donor harbors the F113A mutation and homology arms to the

genomic sequence 5' and 3' of the mutation site. gRNA target sequences and restriction enzyme recognition sites for screening are shown as well as gel electrophoresis of PCR products amplified with indicated primers and cutted by HpyCH4V. A homozygous clone is highlighted in red. (F) TBB treatment reduced the phosphorylation of HP1β by CK2. The construct coding for GFP- HP1β was transfected into HEK293T cells and TBB was directly added into the culture medium. GFP immunoprecipitations performed after expression for 15 hours were visualized by a coomassie blue stained gel. The quantification of phosphorylated to unmodified protein was done with Image J.

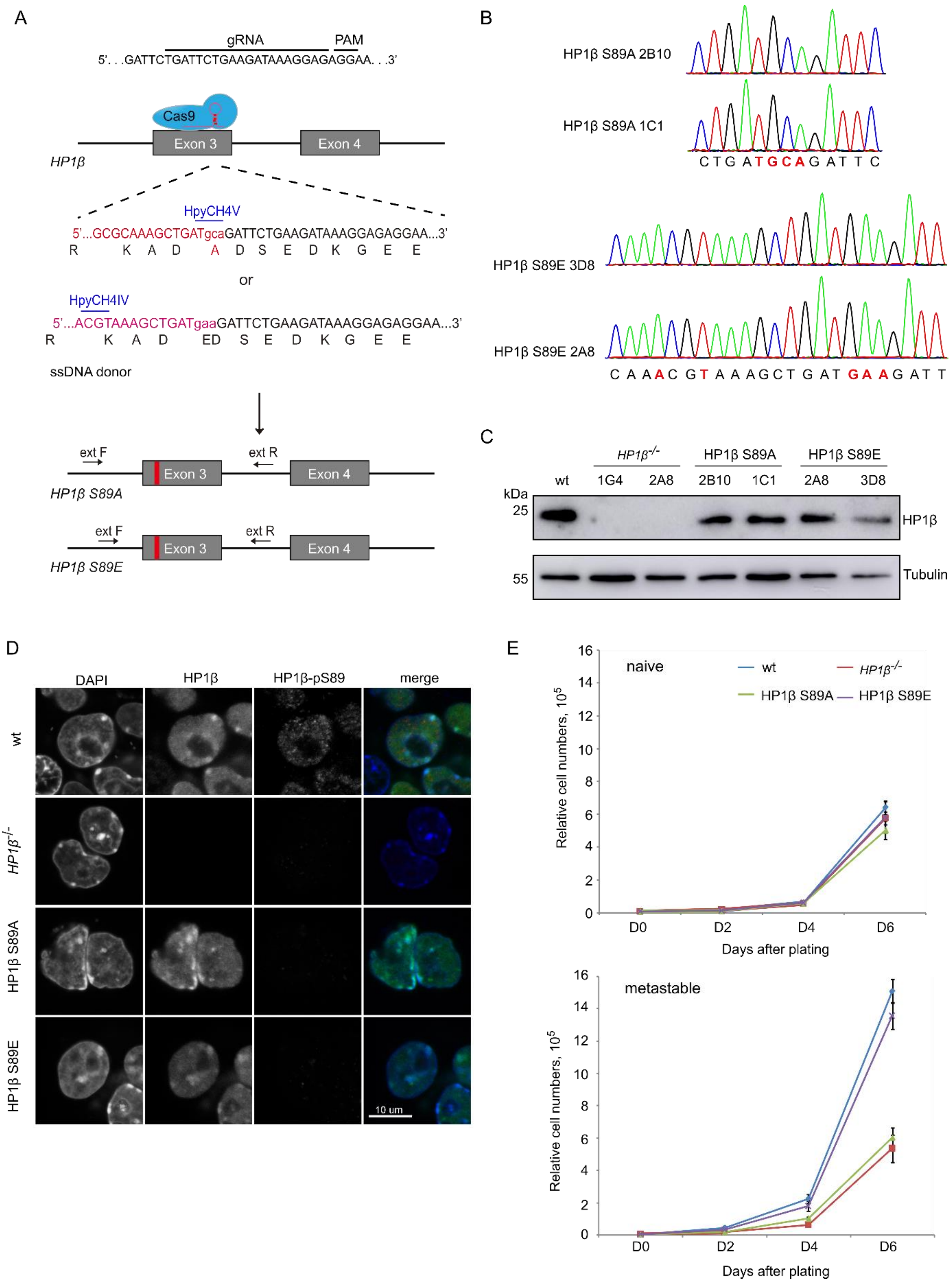

Figure S4. Generation of mESCs carrying different HP1β mutations.
(A) Schematic representation shows the CRISPR/Cas9 gene editing strategy used to generate HP1β S89A and HP1β S89E mESCs. The donors harbor the mutation of S89A or S89E and homology arms to the genomic sequence 5' and 3' of the mutation site. gRNA target sequences and restriction enzyme recognition sites for screening are shown. (B) Amplification

of genomic loci with primers indicated in (A) and confirmation of successful insertion of respective mutations by Sanger sequencing. (C) HP1β level in wt and HP1β mutant cell lines by western blot using a polyclonal HP1β antibody. The tubulin blot was used as a loading control. (D) Immunofluorescence staining *HP1β*$^{-/-}$ and HP1β mutant cell lines using anti-HP1β and anti-HP1β-pS89 antibodies. Scale bar: 10 µm. (E) *HP1β*$^{-/-}$ and HP1β S89A cells proliferate slower than wt E14 and HP1β S89E cells in serum/LIF, but not naïve culture conditions. 5000 mESCs were seeded into 12-well plates in 2i/LIF and metastable mESC medium and counted every second day. Values represent mean ± SEM from four biological replicates.

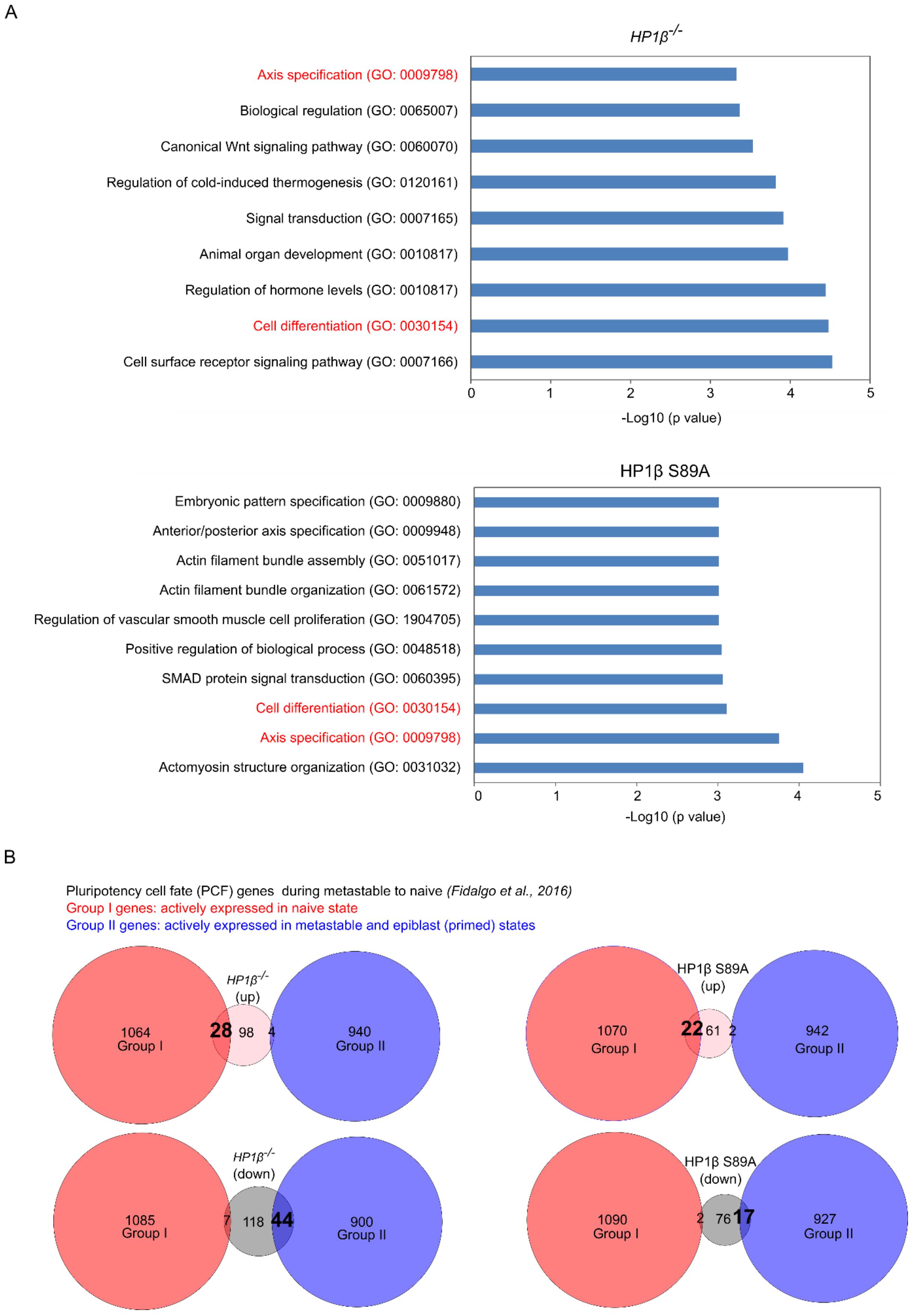
A
HP1β-/-
Axis specification (GO: 0009798)
Biological regulation (GO: 0065007)
Canonical Wnt signaling pathway (GO: 0060070)
Regulation of cold-induced thermogenesis (GO: 0120161)
Signal transduction (GO: 0007165)
Animal organ development (GO: 0010817)
Regulation of hormone levels (GO: 0010817)
Cell differentiation (GO: 0030154)
Cell surface receptor signaling pathway (GO: 0007166)
0 1 2 3 4 5
-Log10 (p value)
HP1β S89A
Embryonic pattern specification (GO: 0009880)
Anterior/posterior axis specification (GO: 0009948)
Actin filament bundle assembly (GO: 0051017)
Actin filament bundle organization (GO: 0061572)
Regulation of vascular smooth muscle cell proliferation (GO: 1904705)
Positive regulation of biological process (GO: 0048518)
SMAD protein signal transduction (GO: 0060395)
Cell differentiation (GO: 0030154)
Axis specification (GO: 0009798)
Actomyosin structure organization (GO: 0031032)
0 1 2 3 4 5
-Log10 (p value)
B
Pluripotency cell fate (PCF) genes during metastable to naive (Fidalgo et al., 2016)
Group I genes: actively expressed in naive state
Group II genes: actively expressed in metastable and epiblast (primed) states
HP1β-/- (up)
1064 Group I
28
98
4
940 Group II
HP1β S89A (up)
1070 Group I
22
61
2
942 Group II
HP1β-/- (down)
1085 Group I
7
118
44
900 Group II
HP1β S89A (down)
1090 Group I
2
76
17
927 Group II

Figure S5. (A) GO analyses were performed using an online tool (http://cbl-gorilla.cs.technion.ac.il/). The GO biological process enrichment of genes with > 1.5-fold change in *HP1β*$^{-/-}$ and HP1β S89A cells. (B) Venn diagrams showing pluripotency cell fate (PCF) genes (Fidalgo et al., 2016) and misregulated genes (>1.5-fold change) in *HP1β*$^{-/-}$ and HP1β S89A mESCs.

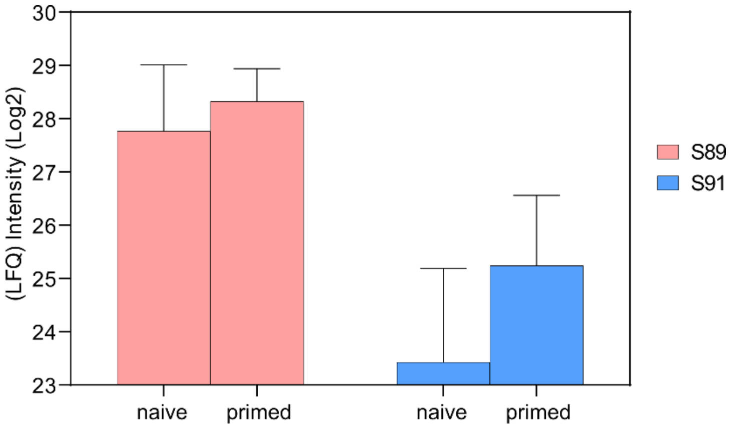

Figure S6. HP1β peptides with S89 and S91 phosphorylation identified by MS analysis during the transition from naïve to epiblast (primed) state (Yang et al., 2019).

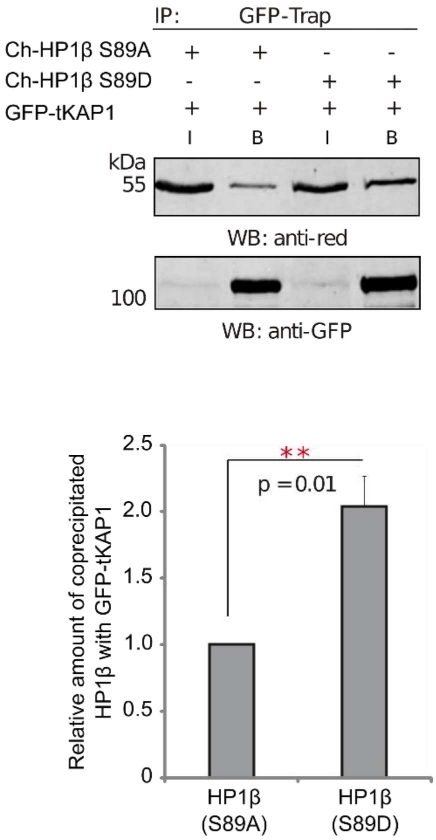

Figure S7. Interaction between HP1β and KAP1

HP1β-pS89 enhances the interaction between KAP1 and HP1β. GFP-KAP1 was immunoprecipitated from HEK293T cells that transiently coexpressed with Cherry tagged HP1β proteins. Bound fractions were separated and visualized with anti-red and GFP antibodies. Intensities of Ch-HP1β S89A and Ch-HP1β S89D in bound fractions were measured with ImageJ and normalized to the corresponding GFP-KAP1 intensity. Values represent mean ± SEM from three biological replicates and the p-value of a two-sided Student’s t-test is indicated.

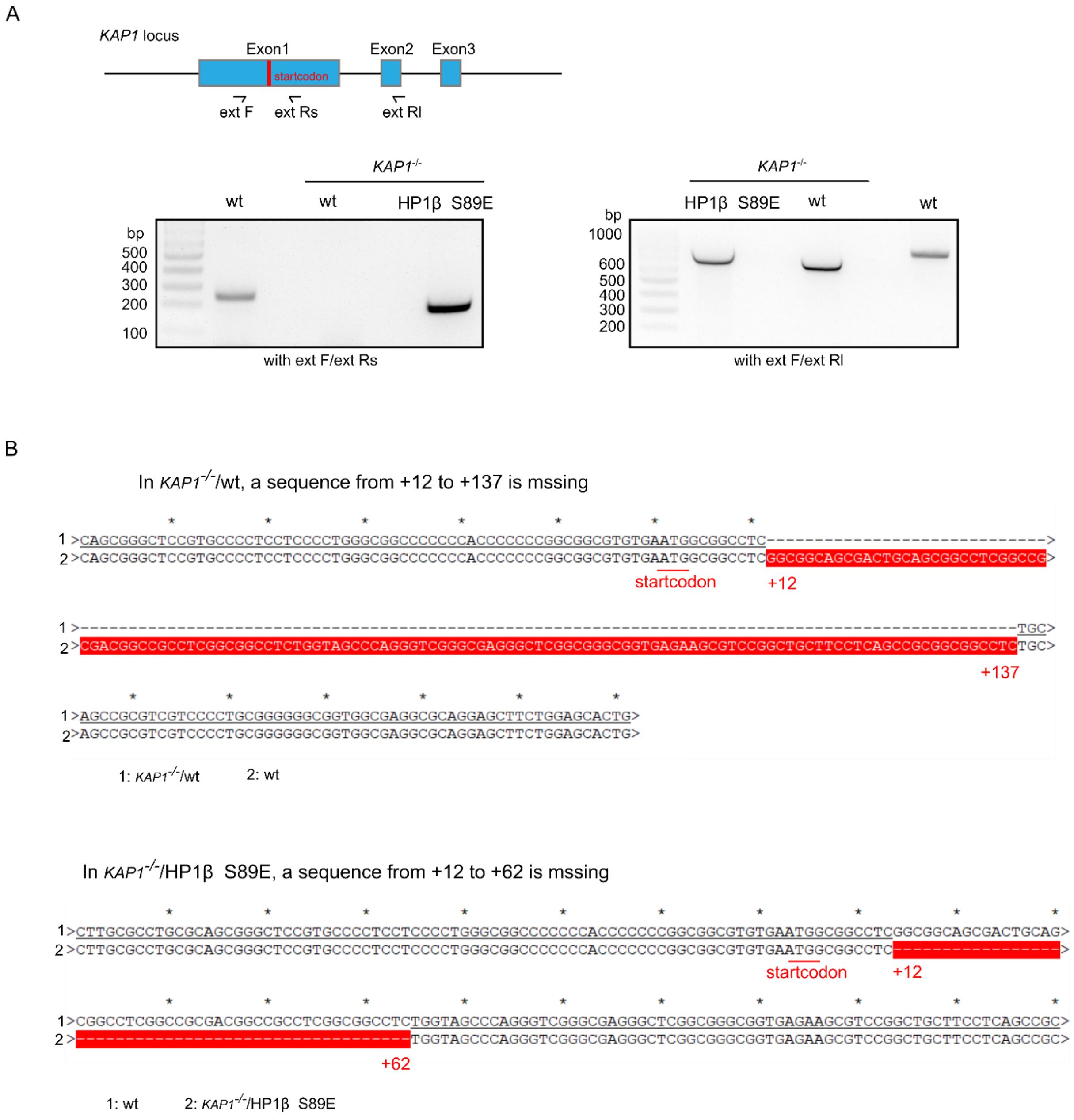

Figure S8. Characterization of *KAP1*$^{-/-}$ cell lines. (A) Gel electrophoresis of the PCR for wt and *KAP1*$^{-/-}$ mESCs with primers as indicated. (B) Alignment of KAP1 locus DNA sequence amplified from wt and *KAP1*$^{-/-}$ cells shows deletions in *KAP1*$^{-/-}$ cells.

A

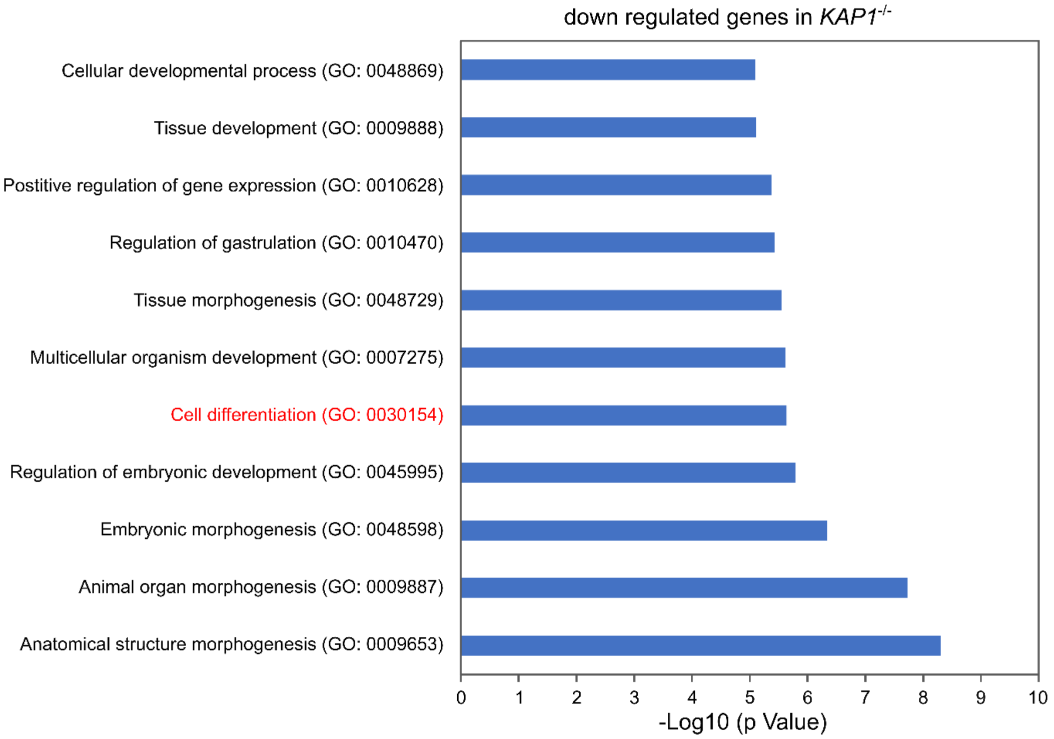

B

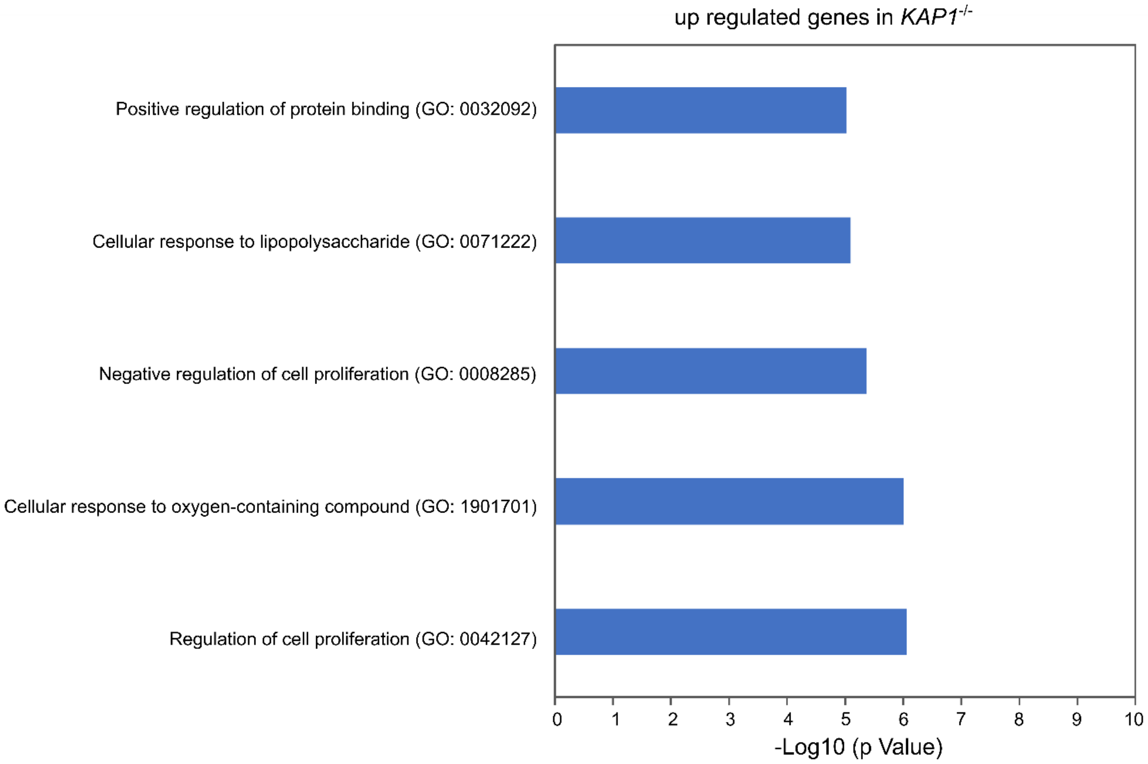

Figure S9. GO analyses were done using an online tool (http://cbl-gorilla.cs.technion.ac.il/). The GO biological process enrichment of genes in *KAP1*$^{-/-}$ (A, downregulated and B, upregulated).

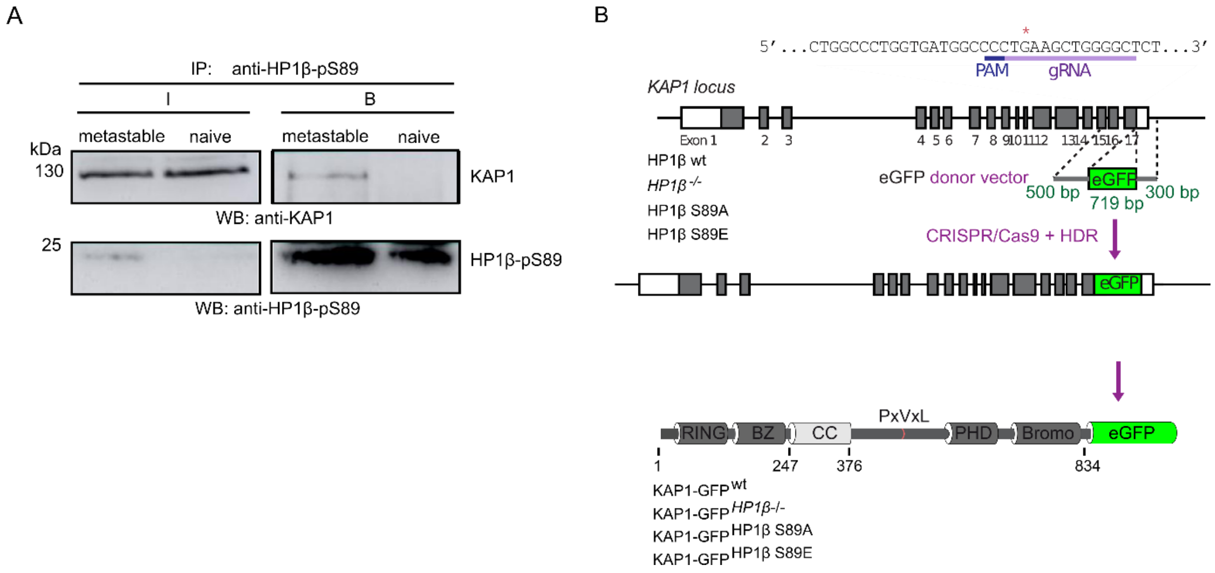

Figure S10. Interaction between HP1β-pS89 and KAP1 and generation of KAP1-GFP knocked in mESCs.

(A) Co-immunoprecipitation shows an interaction between KAP1 and HP1β-pS89 in the metastable but not naive condition. Equal amounts of cell extracts from mESCs cultured in naïve and metastable conditions were incubated with HP1β-pS89 antibodies. Input and bound fractions were analyzed by western blot using HP1β-pS89 and KAP1 antibodies. (B) Schematic representation shows the CRISPR/Cas9 gene editing strategy used to generate KAP1-GFP knockin mESCs with the gRNA target sequence as indicated. The donor harbors the sequence coding for eGFP and homology arms to the genomic sequence 5' and 3' of the respective C-terminal locus.
